# Enhancing CO_2_ Electroreduction Precision to Ethylene and Ethanol: The Role of Additional Boron Catalytic Sites in Cu‐Based Tandem Catalysts

**DOI:** 10.1002/advs.202410118

**Published:** 2024-10-21

**Authors:** Fuqing Yu, Minxing Shu, Guangyao Zhang, Qiming Yu, Hongming Wang

**Affiliations:** ^1^ College of Chemistry and Chemical Engineering Nanchang University Nanchang 330031 China; ^2^ Jiangxi Provincial Key Laboratory of Functional Crystalline Materials Chemistry Nanchang University Nanchang 330031 China

**Keywords:** boron‐doping, electrochemical CO_2_ reduction, multi‐carbon product, tandem catalysts

## Abstract

The electrocatalytic conversion of carbon dioxide (CO_2_) into valuable multicarbon (C_2+_) compounds offers a promising approach to mitigate CO_2_ emissions and harness renewable energy. However, achieving precise selectivity for specific C_2+_ products, such as ethylene and ethanol, remains a formidable challenge. This study shows that incorporating elemental boron (B) into copper (Cu) catalysts provides additional adsorption sites for ^*^CO intermediates, enhancing the selectivity of desirable C_2+_ products. Additionally, using a nickel single‐atom catalyst (Ni‐SAC) as a ^*^CO source increases local ^*^CO concentration and reduces the hydrogen evolution reaction. In situ experiments and density functional theory (DFT) calculations reveal that surface‐bound boron units adsorb and convert ^*^CO more efficiently, promoting ethylene production, while boron within the bulk phase of copper influences charge transfer, facilitating ethanol generation. In a neutral electrolyte, the bias current density for ethylene production using the B‐O‐Cu2@Ni‐SAC0.05 hybrid catalyst exceeded 300 mA cm^−2^, and that for ethanol production with B‐O‐Cu5@Ni‐SAC0.2 surpassed 250 mA cm^−2^. This study underscores that elemental doping in Cu‐based catalysts not only alters charge and crystalline phase arrangements at Cu sites but also provides additional reduction sites for coupling reactions, enabling the efficient synthesis of distinct C_2+_ products.

## Graphical abstract

Incorporating boron (B) into copper (Cu) catalysts enhances the electrocatalytic conversion of CO_2_ into C_2+_ compounds. Boron serves as a supplementary adsorption site for ^*^CO intermediates, promoting ethylene and ethanol production. The B‐O‐Cu2@Ni‐SAC0.05 and B‐O‐Cu5@Ni‐SAC0.2 hybrid catalysts achieve high current densities, showcasing the effectiveness of elemental doping in optimizing selectivity for desirable products.

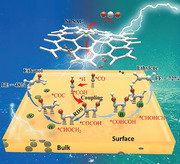

## 1 Introduction

The electrocatalytic conversion of CO_2_ into valuable C_2+_ fuels and chemicals, such as ethylene, acetate, ethanol, and *n*‐propanol,^[^ ^1^ ^]^ holds significant promise due to its potential to mitigate CO_2_ emissions and generate valuable products utilizing renewable electricity.^[^ ^2^ ^]^ The attainment of high selectivity for specific C_2+_ products is imperative for the commercial viability of CO_2_ electrolysis. Copper (Cu) has emerged as a notable electrocatalyst for the efficient conversion of CO_2_ into targeted C_2+_ products, with a particular focus on ethylene and ethanol.^[^ ^3^ ^]^ Nevertheless, the formation pathways for ethanol and ethylene share common intermediates, posing challenges in selectively producing one product over the other.^[^ ^4^ ^]^ Various strategies, including surface species adsorption, facet modulation, vacancy engineering, tuning CO coverage, and elemental doping,^[^ ^5^ ^]^ have been deployed to augment the formation of a single product. However, achieving highly selective product generation through modification of the Cu site, involving charge and conformational complexity alone,^[^ ^6^ ^]^ remains a formidable task, the precise role of the additional introduced elements remains undetermined—whether they function as co‐catalytic sites, charge modulators, or crystalline phase stabilizers.^[^ ^7^ ^]^

To tackle this challenge, our investigation delves into the disparities in the distribution of heterogeneously doped elements in Cu‐based catalysts within the surface and bulk phases, as well as their catalytic advantages in intermediate reaction adsorption, notably ^*^CO. ^*^CO stands as a crucial intermediate in the C_2+_ product generation pathway, and the controlled local concentration of ^*^CO serves to elucidate the catalytic route and extend the catalytic product benefits.^[^ ^8^ ^]^ Consequently, we adopted tandem catalysis, introducing additional sources of CO and customizing Cu‐based substrates to facilitate the direct exposure of various catalytic sites to the reaction intermediates.^[^ ^9^ ^]^ In the generation of C_2+_ products, the reaction pathway bifurcates into CO_2_ conversion to ^*^CO and the subsequent coupling step of ^*^CO.^[^ ^10^ ^]^ However, the catalytic effects of a single site on CO_2_ and CO activation are disparate.^[^ ^11^ ^]^ The tandem catalytic system effectively reduces the energy barrier to CO_2_ activation by manipulating the localized concentration of ^*^CO across a spectrum of tailored Cu‐based catalysts, thereby playing a pivotal role.^[^ ^8^, ^12^ ^]^ This approach concurrently enhances our comprehension of the pathways for ethanol and ethylene product formation.

**Figure 1:**
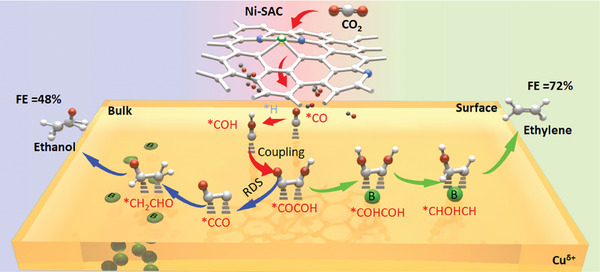
Schematic illustration of catalyst performance and design.

In this study, we synthesized a series of tandem catalysts (B‐O‐Cux@Ni‐SAC) with varying levels of boron (B) doping using nickel single‐atom catalyst (Ni‐SAC) as the CO source. Characterization results confirm that lower B content leads to B enrichment on the catalyst surface, forming atomically dispersed sites. Conversely, higher B content results in B incorporation into the Cu bulk phase, forming a Cu alloy phase. This variance in B content significantly influences the selectivity of the B‐O‐Cux catalyst for ethylene and ethanol products. By adjusting the mass ratio of Ni‐SAC to B‐O‐Cux, we modulated the local CO concentration, thereby inhibiting the hydrogen evolution reaction (HER) and amplifying the product transition. Consequently, B‐O‐Cu2@Ni‐SAC0.05 achieved a Faraday efficiency (FE) of 72% for ethylene, while B‐O‐Cu5@Ni‐SAC0.2 achieved 48% for ethanol at high current densities exceeding 400 mA cm^−2^. Experimental and computational findings indicate that surface B doping provided additional ^*^CO adsorption sites, promoting ^*^CO hydrocoupling to yield ^*^HOCCOH, thereby favoring the ethylene pathway. Additionally, the charge and tensile strain induced by B within the Cu lattice enhanced surface‐bound ^*^CO coverage, consequently reducing the coupling energy barrier and enhancing ethanol selectivity (**Figure** **1**). Disparities in the depth of heterogenous element doping in Cu‐ based catalysts induced product selectivity divergence, ultimately leading to enhanced efficiency of FE in the presence of a single‐atom catalyst. This approach holds promise for the efficient reduction of CO_2_ to the desired C_2+_ product.

## 2 Results and Discussion

### 2.1 Catalyst Preparation and Characterization

**Figure 2:**
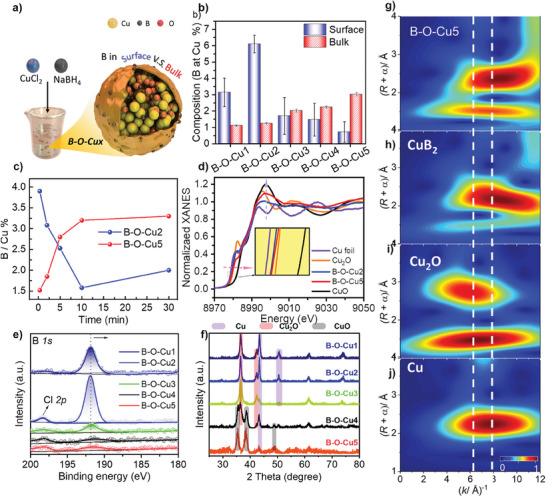
a) Schematic representation of the preparation and components of B‐O‐Cux samples. b) B/Cu wt.% of bulk and surface of B‐O‐Cux samples with different B contents measured by XPS and ICP‐MS. c) Dissolution time‐dependent boron concentration in B‐O‐Cux samples measured by ICP‐MS. d) k‐edge XANES spectra of B‐O‐Cu2 and B‐O‐Cu5 samples. e) Boron XPS spectrum of B‐O‐Cux samples. f) XRD spectrum of B‐O‐Cux samples. g–j) Wavelet transform (WT) –EXAFS Cu K‐edge spectra for B‐O‐Cu5, B‐O‐Cu2, Cu_2_O and Cu foil as a reference. WT maximum positions are indicated as imaginary lines. ‘+α’ indicates that R is not phase‐corrected.

We synthesized B‐O‐Cux samples and characterized their structures. To prepare the B‐O‐Cux catalysts with varying B content, we employed a one‐step wet chemical reduction method (**Figure** **2**a; Figure S2 and S3, Supporting Information).^[^ ^6a^ ^]^ Varying the molar ratio of CuCl_2_ and NaBH_4_ solutions allowed us to control the B content. Inductively coupled plasma mass spectroscopy (ICP‐MS) confirmed the presence of B and its tunability (Table S1, Supporting Information). Interestingly, the B/Cu mass ratio (wt. %) increased in the bulk phase with increasing B content, while the B/Cu mass ratio in the X‐ray photoelectron spectroscopy (XPS) fine spectra showed a different trend, showing a maximum of 5.4 wt. % at B‐O‐Cu2, and low to 0.6 wt. % at B‐O‐Cu5 (Figure 2b; Figure S4, Supporting Information).^[^ ^5b^ ^]^ We also synthesized B‐free Cu_H_ catalysts to compare the B doping effect (Figure S5, Supporting Information). Taking into consideration that merely changing the type of reducing agent did not yield crystal sizes akin to B‐O‐Cux, we synthesized a comparative sample, Cu_H_‐1, and presented its TEM images alongside CO_2_RR performance (Figures S6 and S7, Supporting Information). In order to accurately measure the bulk‐phase boron concentration of B‐O‐Cux samples, we measured the dissolution time‐dependent boron concentration, whose depth of detection was positively correlated with the dissolution time in dilute nitric acid solution indicating surface enrichment of B at lower B doping levels and subsidence into the Cu bulk phase at higher B levels (Figure 2c; Table S2, Supporting Information).^[^ ^13^ ^]^

Similarly, XPS was used to study the oxidation state and chemical composition of the sample surface.^[^ ^14^ ^]^ In the corresponding B 1s spectra (Figure 2e), an expected increase in B binding energy with increasing B content was observed. However, it was also accompanied by a relative decrease in the surface B peak with increasing B content. In the Cu 2p_3/2_ fine XPS (Figure S4b, Supporting Information), an attributed peak of CuO is observed at ≈933.7 eV. The Cu 2p and B 1s spectra both indicate their presence in a deficient electron state, thereby suggesting the predominance of oxide states on their surfaces, since both Cu and B are oxide species, they might be susceptible to invasion by oxygen from the air. Similarly, the powder X‐ray diffraction (XRD) patterns exhibit a transition from the Cu crystalline phase to the Cu_2_O crystalline phase, and eventually to the CuO crystalline phase with increasing B content (Figure 2f).^[^ ^15^ ^]^ This trend aligns with the increasing oxidation states of Cu, where the primary form of Cu^σ+^ exists as CuO_x_ (since B also exists as BO_x_). Given that only the amount of CuCl_2_ was varied and the samples were obtained under highly reducing conditions, we believe that all samples were initially predominantly in the metallic state and were rapidly oxidized to oxides after the reaction. The fundamental reason affecting the oxidation states post‐reaction is the grain size. As shown in Figure 2f, with increasing B content, the broadening of diffraction peaks indicates smaller grain sizes. Additionally, as shown in Figures S8 and S9 (Supporting Information), the oxidized B‐O‐Cu5 corresponds to a larger specific surface area. Additionally, a B‐deficient CuH was synthesized. XRD analysis revealed that the Cu phase closely resembled the B‐O‐Cu1 sample with lower B content in bulk phase, consisting of Cu_2_O and minor Cu phases (Figure S10, Supporting Information).

We conducted analyses utilizing X‐ray Absorption Near‐Edge Structure (XANES) as depicted in Figure 2d.^[^ ^16^ ^]^ The determination of the peak white line position at the Cu K‐edge corroborates the Cu valence state, which spans between 0 and 1. Also, the Cu K‐edge white line manifests at values encompassing this range. Upon closer examination of the edge front data, a discernible rise in the valence state of B‐O‐Cu5 in comparison to B‐O‐Cu2 emerges, the assertion aligns with the findings illustrated in the near‐edge first‐order derivative plot of the Cu K‐edge (refer to Figure S11, Supporting Information). Based on XRD and XPS data, the crystalline phases of B‐O‐Cu5 are manifested as CuO and Cu, but in the regionally scanned valence states of B‐O‐Cu5, the valence states are between 0 and 1, indicating the heterogeneity of the B‐O‐Cux structure. The XANES spectra of B‐O‐Cu5 show ≈+1 valence state, so the introduction of more B makes the local regions more susceptible to invasion by O species, resulting in an increase in local valence states, subsequently leads to inconsistencies in the local valence states within the structure.

Utilizing high‐resolution wavelet transform (WT)‐ Extended x‐ray absorption fine structure(EXAFS) analysis in both R‐space and K‐space (as illustrated in Figure 2g–j), discernible peaks of Cu bonding to lighter elements were identified within the lower K‐space. Notably, B‐O‐Cu5 exhibited a larger region compared to B‐O‐Cu2 in the lower K‐space. Considering the preceding XRD patterns, it can be inferred that while the Cu crystal phase remains the predominant crystalline phase of B‐O‐Cu2, the introduction of B induces localized high oxidation from the air, leading to a change in the overall valence state of B‐O‐Cux. Moving into the high K‐space, the observed increase in the K value of the Cu─Cu coordination center indicates an increase in the bond energy of the Cu─Cu bonds in B‐O‐Cux. This shift signifies an augmented Cu─Cu bond energy, electron transfer to the surroundings, and an escalated oxidation state (Figures S12 and S13, Supporting Information).

A higher B content appears to favor the existence of Cu^σ+^ in this compound. Due to the predominant coordination of Cu with O species, the introduction of B merely facilitates additional adsorption sites for O species, we utilize the Cu─Cu path of Cu and the Cu─O path of Cu_2_O to hypothesize the coordination environment around Cu. Analysis of the fitting outcomes from B‐O‐Cu2 to B‐O‐Cu5 reveals a progressive increase in the Cu─O coordination from 1.29 to 1.44, accompanied by a decrease in the coordination number of Cu─Cu from 2.33 to 1.92 (Tables S4 and S5, Supporting Information). These findings strongly suggest that the Cu─Cu coordination within the B‐O‐Cu5 samples experiences congestion due to the presence of lighter elements, thereby influencing an elevation in the oxidation state.

**Figure 3:**
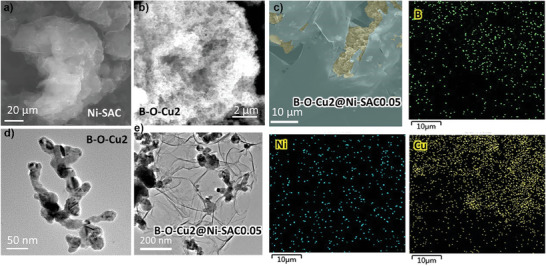
SEM images of a) Ni‐SAC, b) B‐O‐Cu2. c) EDS maps of the corresponding Cu, B. Ni elements of the catalysts after hybridization. d, e) TEM image of B‐O‐Cu2 and Hybridized catalyst, respectively.

We utilized a previously synthesized Ni‐SAC catalyst with the “Ni‐N_2_C_2_” configuration, known for its robust CO_2_ catalytic performance under neutral conditions.^[^ ^17^ ^]^ High‐angle annular dark‐field scanning transmission electron microscopy (HAADF‐TEM) images provided confirmation of the monodispersion of Ni atoms in the Ni‐SAC (see Figure S1, Supporting Information). In contrast, B‐O‐Cu2 displayed a distinctive porous morphology, featuring a specific surface area (S_BET_) of 24.5 m^3^ g^−1^, as measured in Figure S8 (Supporting Information). This porous structure exhibited a prominent hysteresis loop curve, indicative of the presence of micropores and mesopores ≈1.3 and 100 nm, respectively (refer to **Figure** **3**b). Notably, Ni‐SAC displayed a thin sheet with an interlayer convolutional morphology (as observed in Figure 3a). Subsequently, the B‐O‐Cux catalyst was dissolved in a methanol solution with Ni‐SAC catalyst, followed by ultrasonication for 0.5 h, yielding the hybrid catalyst B‐O‐Cux@Ni‐SACy, where y denotes the mass ratio of B‐O‐Cux sample to Ni‐SAC catalyst. The energy‐dispersive spectroscopy (EDS) elemental mapping of the hybrid catalyst confirmed the independent distribution of Cu and C elements, indicating the separation of the two phases, B‐O‐Cux and Ni‐SAC (Figure 3c; Figures S14 and S15, Supporting Information). And the elemental mapping and content ratios of the high‐resolution TEM of the B‐O‐Cu2 samples are in Figure S16 (Supporting Information). However, the B element mapping is close to the Be window, and therefore does not represent the actual B element content. The morphology of B‐O‐Cux remained largely unaltered both before and after assembly (as depicted in Figure 3d,e).

### 2.2 Performance for CO‐to‐Ethanol and Ethylene Electroreduction

**Figure 4:**
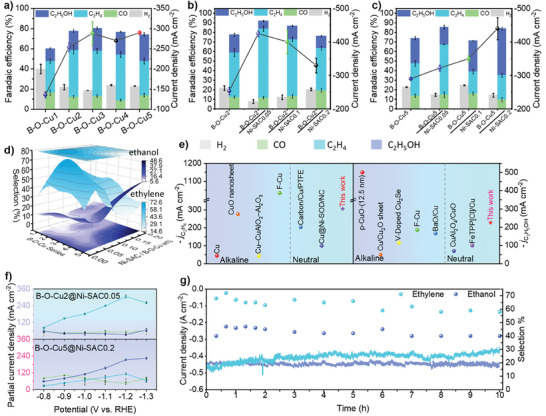
Electrochemical CO_2_ reduction reaction (CO_2_RR) performance. Faradaic efficiencies (FEs) of major CO_2_RR products obtained on a) B‐O‐Cux samples with different B contents, b,c) B‐O‐Cu2 and B‐O‐Cu5 samples hybridized into different ratios of Ni‐SAC at −1.2 V vs. RHE. d) 3D surface plot of the Fes of B‐O‐Cux samples hybridized into different ratios of Ni‐SAC at −1.2 V vs. RHE. e) Compare this work with related electrocatalysts in terms of electrolyte acidity and alkalinity, ethane, and ethanol bias current density. f) Bias current density at different bias voltages for the two samples with the best FEs. (The upper part is the ethylene bias current; the lower part is the ethanol bias current, the cyan line – ethylene, the blue – ethanol, the gray – H_2_, and the green – CO) g) Chronoamperometric *i*–*t* curve (left axis) and the corresponding FE_C2+_ (right axis) for B‐O‐Cu2@Ni‐SAC0.05 and B‐O‐Cu5‐@Ni‐SAC0.2 at −1.2 V vs. RHE during a testing period of 10 h.

The CO_2_RR performance of the synthesized catalysts was tested in a flow cell in 0.5 m KHCO_3_ electrolyte (Figure S17, Supporting Information). Gas chromatography (GC) analysis revealed the presence of H_2_, CO, methane (CH_4_), and ethylene (C_2_H_4_), while ^1^H‐NMR analysis detected methanol (CH_3_OH), ethanol (C_2_H_5_OH), and n‐propanol in the liquid phase products (Figures S18–S20, Supporting Information), considering the relatively low selectivity of formate and acetate products, they have been excluded from the FE chart to enhance the contrast of product conversion. Initially, we investigated the effects of different B doping levels on the CO_2_RR performance (**Figure** **4**a). Among the catalysts tested, B‐O‐Cu2 demonstrated the highest C_2+_ faradaic efficiency (FE) of 67% at a voltage of −1.2 V vs. RHE, with a current density exceeding 250 mA cm^−2^.^[^ ^18^ ^]^ B‐O‐Cu2 and B‐O‐Cu5 exhibited specific ethylene‐ethanol product transition patterns, resulting in a reduction in the FE of C_2_H_4_ from 46% to 34% and an increase in ethanol selection by 10%. These observations echoed the differences in B‐element content between the surface and bulk phases of B‐O‐Cu2 and B‐O‐Cu5, as characterized earlier. To enhance this effect, we prepared hybrid catalysts with different ratios of Ni‐SAC to B‐O‐Cux (Figure 4b), achieving ethylene yields of up to 72% at a Ni‐SAC to B‐O‐Cu2 mass ratio of 0.05, at −1.2 V vs. RHE, with a maximum current density close to 450 mA cm^−2^. Ni‐SAC was used as a substrate as well as an indirect source of CO, and the separate reduction product monitoring of Ni‐SAC showed only H_2_ and CO, the latter with a Faraday efficiency (FE) of 85 ± 1% at −1.2 V vs. RHE and a partial current density of 120 mA cm^−2^ (Figure S21, Supporting Information).

To study the reaction kinetics of the catalyst, Nyquist plots were measured at open‐circuit potential. Electrochemical impedance spectra (EIS) analysis (Figure S22, Supporting Information) revealed that the addition of Ni‐SAC effectively reduced the charge transfer impedance (R_ct_) at the operational voltage. Considering the reactant mass transfer equation Z_Re_ = R_Ω_ + R_ct_ – 2σ^2^C_d_, where σ represents the mass transfer rate from the reactant to the reaction interface during the reaction process, the indirect CO generation by adding Ni‐SAC improves the transfer efficiency of CO diffusion to the Cu reaction interface.^[^ ^19^ ^]^ Furthermore, we measured the electrochemically active surface area (ECSA) of the catalysts, B‐O‐Cu2 doped with a small amount of B exhibited the highest Double Layer Capacitance Values (C_dl_) of 5.93 mF cm^−2^, higher than that of the Cu_H_ catalyst, suggesting the possibility that B dissolves in Cu and participates in CO_2_RR. In addition, the C_dl_ of B‐O‐Cu5 increased greatly from 3.13 to 6.96 mF cm^−2^ after the addition of Ni‐SAC catalysts, indicating that the homogeneous dispersion of B‐O‐Cux by the carbon carriers effectively increased the reaction area of the catalysts, which led to the improvement of the mass transfer rate and the expansion of the active surface area (Figure S23, Supporting Information).

Despite a decrease in current density and FE_C2+_ yield with increased Ni‐SAC content impregnated with B‐O‐Cu2, the biased generation trend for C_2_H_5_OH and CO products remained consistent. We conducted coordinated CO_2_RR testing of B‐O‐Cux and Ni‐SAC ratios with different mass fractions of 0.05, 0.1, and 0.2. Surprisingly, the different Ni‐SAC ratios amplified the tendency for ethanol‐ethanol product differentiation, with the highest ethylene selectivity of 72% observed for B‐O‐Cu2@Ni‐SAC0.05 and 46% for B‐O‐Cu5@Ni‐SAC0.05 (Figure 4d,f; Figure S24–S27, Supporting Information). Compared to the Cu_H_ catalyst without B, the current density and C_2+_ selectivity were significantly improved (Figure S28, Supporting Information). Further product distribution analysis indicated that in the hybridized Ni‐SAC system, FEs of H_2_ were significantly lower compared to the unhybridized condition, while FEs of CO were elevated and the catalytic activity of CO_2_ was enhanced (Figure 4c; Figure S29, Supporting Information). This suggests that the tandem system of indirectly generated CO inhibits the hydrogen evolution reaction (HER) and reduces the reactive energy barriers for CO generation. Moreover, the results of FEs ratios of C_2+_/CO indicate that a majority of the formed ^*^CO is inclined to participate in the C–C coupling process, promoting sustained generation of ethylene products with a specific localized CO coverage. However, with a continuous increase in the CO source hybridization ratio, CO conversion ability weakened, whereas B‐O‐Cu5 in a higher local CO environment favored the ethanol differentiation pathway. Figure 4f shows the bias current densities of the best two samples at different bias voltages. We conducted tests on the FE of CORR for B‐O‐Cu2 and B‐O‐Cu5 to further validate the influence of CO on the performance of B‐O‐Cux catalysts. Both exhibited high selectivity toward ethylene at lower potentials, indicating that direct CO involvement contributes to achieving C–C coupling at lower overpotentials for B‐O‐Cux catalysts. At −0.6 V vs. RHE, B‐O‐Cu2 displayed significantly higher ethylene selectivity (≈60%) compared to B‐O‐Cu5 (40%), highlighting the advantageous pathway for ethylene differentiation. Although B‐O‐Cu5 yielded 16% ethanol, surpassing B‐O‐Cu2, ethanol production did not dominate. This suggests that CO concentration and subsequent hydrogenation or hydroxylation processes play crucial roles in product differentiation (Figure S30, Supporting Information).

Therefore, we aimed to evaluate the significance of the individual roles of Ni‐SAC and B in product conversion. As shown in Figure S31a (Supporting Information), the trend of the ethylene/ethanol conversion ratio across different B‐O‐Cux samples remains almost consistent, indicating that the individual role of Ni‐SAC is similar across different samples, functioning as a CO source and providing localized CO concentration. Additionally, B‐O‐Cu3‐5 samples did not exhibit a significant response to varying Ni‐SAC loadings, suggesting the inertness of Ni‐SAC in product differentiation. However, B‐O‐Cu1‐2 displayed high ethylene conversion rates, indicating that different samples respond to localized CO concentration to varying degrees. In Figure S31b (Supporting Information), under a 5% wt. Ni‐SAC loading, we correlated the FE ratio of the products with the surface B/Cu atomic ratio from previous literature. The results indicate that an increased surface B atomic ratio enhances ethylene selectivity. Therefore, we infer that B atoms can act as reaction participants, affecting CO adsorption capability, and ultimately influencing the selective formation of the final products. Additionally, an optimal amount of Ni‐SAC can act as a CO source to promote the C–C coupling pathway, while excessive Ni‐SAC may limit complete CO conversion. Based on the above catalytic evaluations of the two phases, we believe that boron atom concentration can be considered the primary factor enhancing ethanol/ethylene production.

To evaluate the CO_2_RR performance, we compared the performance of the B‐O‐Cux@Ni‐SACy catalyst with other top catalysts reported in the literature (Figure 4e).^[^ ^5^, ^8^, ^11^, ^20^ ^]^ Regarding the selectivity for ethylene production, the B‐O‐Cu2@Ni‐SAC0.05 catalyst exhibits one of the best catalytic performances under neutral conditions, second only to the F‐Cu catalyst under alkaline conditions. In terms of partial current density for ethanol production, B‐O‐Cu2@Ni‐SAC0.05 is among the best catalysts reported to date. The stabilized CO_2_RR properties of B‐O‐Cu2@Ni‐SAC0.05 and B‐O‐Cu5@Ni‐SAC0.05 were tested at a constant voltage of −1.2 V vs. RHE. As shown in Figure 4g, the bias current densities of the products remained above 200 mA cm^−2^ for 10 h. The stability *i‐t* test of B‐O‐Cu2@Ni‐SAC0.05 for a total of 3 h (2h of operation) was tested by the pulse voltage method (where −1.2 V vs RHE is the operating voltage and −0.02 V vs RHE is the regeneration voltage), which showed a reduction in carbonate precipitation and no change in the anode electrolyte, suggesting that carbonate generation is a subsequent selectivity and stability degradation Key factors (Figure S32, Supporting Information). The morphology of the catalysts remained largely unchanged after a long period of CO_2_ electrolysis (Figure S33, Supporting Information). In summary, the B‐O‐Cux@Ni‐SACy catalyst shows great promise for the conversion of CO_2_–C_2+_ products.

### 2.3 In Situ Characterization of the B‐O‐Cux

**Figure 5:**
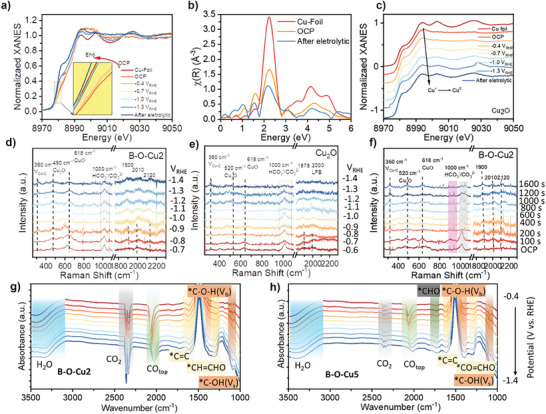
a) In situ k‐edge XANES spectra of B‐O‐Cu2 with varying application voltages (The inset shows the zoomed‐in pre‐edge peak data). b) Comparison of R‐space data before and after the reaction. c) K‐edge XANES spectra of Cu_2_O samples after being electrochemically reduced. In situ Raman spectra of the d) B‐O‐Cu2 and e) Cu_2_O samples working at different potentials in 1 m KHCO_3_. f) Time‐dependent in situ Raman spectra of B‐O‐Cu2 samples at −1.2 V vs. RHE. g,h) In situ ATR‐FTIR spectra of B‐O‐Cu2 and B‐O‐Cu5 from 2500–1000 cm^−1^ working at different potentials in 0.5 m KHCO_3_. OCP means open circuit potential.

**Figure** **5**a shows that B‐O‐Cu2 remained stable when the voltage was applied from the open‐circuit voltage (OCP) to −1.3 V vs. RHE. The K‐edge XANES analysis of Cu revealed a decreasing trend in the valence state as the voltage was continuously decreased. However, there was almost no significant change after reaching −1V vs. RHE. This suggests that the structure stabilized at the optimal reduction potential voltage, and the final valence state was slightly higher than Cu^0^. This could be due to the stabilizing effect of B in the catalytic system. Based on the first‐order derivative mapping of K‐edge XANES (Figure S34, Supporting Information), we can infer that the valence state of Cu changed less throughout the reaction and no new peaks emerged. Additionally, we conducted ICP and XPS tests on B‐O‐Cu2 and B‐O‐Cu5 after the reaction. The results indicate that the relationship between the surface and bulk phases of B remained unchanged from before the reaction. However, the ratio of elemental B on the surface increased after the reaction, suggesting that elemental B tended to dissolve to the surface during the reaction (Figures S35 and S36 and Table S3, Supporting Information). After completing the reaction, we left it in the air for 30 s. The final valence state of Cu was almost fully oxidized to the OCP state, while the height of the white line peak was slightly lower than that at the OCP. This may be due to the intrinsic property of the nanoparticle being highly susceptible to oxidation. The R‐space maps before and after the reaction indicate that the structure of the B‐O‐Cu2 samples remained largely unchanged, with the bulk phase still dominated by the Cu crystalline phase coordinated with light elements (Figure 5b). In comparison to in situ experiments with commercial Cu (Figure 5c), the B element did exhibit a tendency to dissolve out to the surface during the reaction while maintaining the overall structure and valence stability.

In situ, Raman measurements were conducted to investigate the conformational and phase changes of the B‐O‐Cu2 catalyst during the CO_2_RR process.^[^ ^20g^ ^]^ The spectrum of B‐O‐Cu2 was tested under different applied voltages, revealing a continuous increase in the peak at 360 cm^−1^,^[^ ^21^ ^]^ which is attributed to the Cu─C bond, indicating the accumulation of Cu‐adsorbed carbon intermediates as the voltage increases. Two peaks were observed at ≈490 and 618 cm^−1^ (Figure 5d), corresponding to the characteristic Cu─O─B and CuO bonds, respectively. In Figure 5e, the standard Cu_2_O sample shows characteristic peaks at 520 and 620 cm^−1^ for Cu_2_O and CuO, respectively, suggesting that the initial state of the Cu‐O‐B2 sample contains a higher CuO oxidation feature. Under subsequent high‐potential conditions, the Cu^+^ peaks of the Cu_2_O sample were continuously reduced, with a minor Cu‐OH peak appearing near 532 cm^−1^. In contrast, in the Cu‐O‐B2 sample, the Cu_2_O characteristic peak was obscured, leaving only the vibrational peak of Cu‐O‐B at 490 cm^−1^. This observation is corroborated by existing Raman studies but be overlooked,^[^ ^6a^ ^]^ and demonstrates the retention effect of boron on oxygen, which promotes local electronic state stability. As the reaction voltage was prolonged, Raman signals of adsorbed CO were observed at ≈2000 cm^−1^. These signals mainly consisted of bridged adsorption (^*^CO_B_) at 1900 cm^−1^, top adsorption (^*^CO_L_) at 2000 cm^−1^, and an additional CO adsorption peak (^*^CO_E_) at 2100 cm^−1^.^[^ ^22^ ^]^ In the voltage‐dependent spectra of the control Cu_2_O samples (Figure 5e), the Cu^σ+^ oxidation peak was significantly reduced at the initial −0.8 V vs. RHE, and almost disappeared after −1.0 V vs. RHE. By contrast, the B‐O‐Cu2 catalyst basically achieved Cu^σ+^ stabilization under electrolytic reaction. In the region of the ^*^CO adsorption peak, only the adsorption peak type of ^*^CO_B_ and ^*^CO_L_ was present for Cu_2_O, and no ^*^CO adsorption peak at 2100 cm^−1^ was observed, indicating the presence of additional ^*^CO adsorption sites, which is therefore presumed to be related to element B. Additionally, Cu_2_O exhibited higher adsorption of CO at lower voltage. In contrast, the spectra of B‐O‐Cu2 consistently show a strong adsorption peak for *CO, indicating that ^*^CO intermediates are retained more easily in B‐O‐Cu2 samples. This correlates with both the stabilization of Cu^σ+^ and the presence of additional adsorption sites. The time‐dependent in situ Raman spectra of B‐O‐Cu2 were tested at 1.2 V vs. RHE (Figure 5f). The CuO*_x_* peak at 628 cm^−1^ indicated a slight decrease in Cu^σ+^ valence during 200 s of sustained electrolysis, but remained steady throughout the process after 200 s. The Cu‐C peak at 360 cm^−1^ continued to elevate, indicating the accumulation of hydrocarbon products over time. At the Raman peak between 800 and 1000 cm^−1^, a persistent broad peak is observed, which may be related to Cu─B bonding. This peak can be roughly matched with the solid‐state Raman spectrum (Figure S37, Supporting Information), but no relevant literature about the Raman signal was accurately reported. In the region of ^*^C═O adsorption peaks, after 100 s of electrolysis, ^*^CO_B_,^*^CO_L_, and ^*^CO_E_ appeared, of which ^*^CO_L_ and ^*^CO_E_ were the main adsorption peaks during the reaction process, suggesting that the B site may act as an additional ^*^CO_E_ adsorption site.

To comprehend the disparate CO_2_RR performance of B‐O‐Cu2 and B‐O‐Cu5 catalysts, we employed surface‐enhanced Fourier Transform InfraRed‐based (FTIR) spectroscopy to identify crucial adsorbed intermediates within the electrocatalytic process.^[^ ^6^, ^23^ ^]^ We utilized the ─OH stretching vibrational peaks of H_2_O under nearly identical solution conditions within the reaction as our reference for assessing the intensity of the intermediates. In Figure 5g,h, we observed the corresponding CO_2_ adsorption peaks at 2360 cm^−1^ for both samples, and the one for B‐O‐Cu5 is significantly weaker than that for B‐O‐Cu2, which indicates the rapid conversion of CO_2_ on B‐O‐Cu5. The absorption bands at 2050 cm^−1^ correspond to surface‐bound C≡O (^*^CO) and at 1723 cm^−1^ correspond to the hydrogenation intermediate on C of ^*^CO–^*^CHO. Significantly, the higher ^*^CO peak mirrors that of B‐O‐Cu2 indicating additional ^*^CO sites and that the conversion of ^*^CO on B‐O‐Cu5 is more favorable to ^*^CHO. The stretching vibrations (V_s_) at 1610 cm^−1 *^C═C, 1290 cm^−1 *^CO═CHO indicate that both samples have the same reaction intermediates ^*^CO═CHO in their respective reaction pathways. However at 1084 cm^−1 *^C─OH (V_s_) and the bending vibration (V_b_) of ^*^C─O─H at 1390 cm^−1^, B‐O‐Cu2 and B‐O‐Cu5 exhibited similar Vs for ^*^C─OH, while the V_b_ of B‐O‐Cu5 is stronger than B‐O‐Cu2′s, We hypothesize that the vibrations of ^*^COH originate from different reaction intermediates ─^*^H_3_CCH_2_OH and ^*^COCOH. Since the former is the signature peak for ethanol production and ^*^COH is the O hydrogenation intermediate of ^*^CO, the bending vibrational peak of the former exhibits a stronger peak. In summary, the reaction pathway of B‐O‐Cu5 favors the ^*^CHCHO pathway, with the signature ^*^COH bending vibrational peak of ethanol revealed. The B‐O‐Cu2 sample, on the other hand, shows the coexistence of ^*^CHCHO and ^*^CHCOH in the reaction, which may point to the presence of multiple sites in the reaction, and the more intuitive proton‐coupled electric transfer (PCET) process needs to be verified by further simulation calculations.

### 2.4 Theoretical Calculations

**Figure 6:**
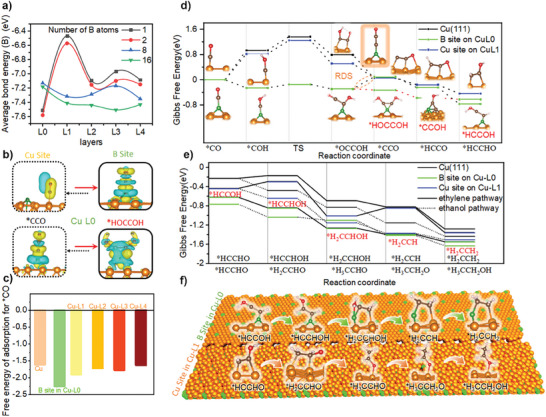
a) Average bond energies of individual B atoms corresponding to the layers of the Cu lattice when different numbers of B atoms are inserted through them. b) Gibbs free energy step diagram for the transition of ^*^co to ethylene ethanol differentiation intermediates (^*^HCCOH and ^*^HCCHO). c) The Gibbs free energy of adsorption of ^*^CO at the B site and the Cu site changes when a B atom is inserted into the interlayer of the crystalline phase of Cu as well as without the addition of B. d) Electron density difference plots for the Cu‐B bonding configuration and when it adsorbs ^*^CO. e) The Gibbs free energy step diagrams for final C_2+_ product differentiation at the B site of Cu‐L0, at the Cu site of Cu(111), and of Cu‐L1. f) The optimal ethylenic ethanol product differentiation routes and atom migration steps.

We employed first‐principles Density Functional Theory (DFT) calculations to investigate the structural stability of B atoms within the bulk phase of Cu. These computations were based on experimentally determined variations in B content under strongly reducing conditions (**Figure** **6**a). Specifically, we introduced 1, 2, 8, and 16 B atoms into discrete layers of the Cu (4 × 4 × 6) crystalline phase and subsequently determined the optimized geometries, as depicted in Figures S38 and S39 (Supporting Information). Remarkably, the atomic radii of B are considerably smaller than those of Cu. Computational analyses reveal that B atoms are densely clustered and distributed within the layers of the Cu crystalline structure. Subsequent to these optimizations, we conducted bond energy calculations for the B atoms. Figure 6a showcases the average bond energies of individual B atoms in various layers within the crystalline phase and sheds light on the optimal structural configurations of B atoms at different B contents. Consistent with the experimental findings, at low B atom concentrations, the atoms predominantly adhere to the surface. Conversely, at higher B atom concentrations, clusters are distributed within the bulk phase of Cu.

Based on the experimental results of the changes in product selectivity associated with different levels of B distribution within the Cu crystalline phase, we designed three catalytic configurations; Cu(111), Cu‐L0 (B‐O‐Cu2), and Cu‐L1 (B‐O‐Cu5). Considering the low boron content and surface enrichment in Cu‐O‐B2, Cu‐L0 was selected to represent the Cu‐0‐B2 sample. While the sampling depth of XPS is 3–5 nm, Cu‐L1 was chosen to represent the Cu‐O‐B2 sample to account for boron's role in influencing electronic configuration rather than acting as a reactive site. The structural configurations of both Cu and Cu‐L(0‐5) are also listed in Figure S40 (Supporting Information). According to the free energies of individual B atoms calculated in Figure 6a, Cu‐L0 is the introduction of individual B atoms on the surface of the crystalline phase of Cu(111), and Cu‐L1 is the insertion of eight B atoms through the L1 layer of Cu(111). We then performed first‐principles DFT calculations to explore the pathways of C_2+_ product formation. Previous DFT studies have emphasized the efficacy of the Ni‐N_2_C_2_ SAC in converting CO_2_–CO products.^[^ ^17^ ^]^ Therefore, we first discuss the unique adsorption properties of ^*^CO on these three catalytic configurations. As shown in Figure 6c, the Gibbs free energy of ^*^CO adsorption on Cu(111) was −1.605 eV. When a single B atom is introduced on the Cu‐based surface, the differential density of adsorption charge on the Cu site is concentrated on ^*^CO, which is unfavorable for the retention of ^*^CO, while the B site exhibits strong adsorption of ^*^CO, which is lower than the Gibbs free energy of ^*^CO adsorption on Cu(111) of 0.65 eV (Figure 6b). Therefore, the B site on Cu‐L0 can be used as an additional adsorption site for ^*^CO for subsequent C_2+_ product conversion.

To delve further into the impact of B on Cu sites within the Cu crystalline phase, we analyze the pathway from ^*^CO to the intermediate ^*^HCCHO (Figure 6d). In previous calculations, we addressed the sequential problem of proton charge transfer, manifesting as the optimal reaction pathway involving H addition followed by transition state (TS) coupling.^[^ ^24^ ^]^ Notably, the C═C coupling process, being heat‐absorbing and influenced by external factors, serves as the rate‐determining step (RDS) in this system. The free energy of this coupling process on the Cu site changes from 0.43 to 0.42 eV with the introduction of higher B content, indicating that ^*^CO is more likely to generate C_2+_ products on Cu sites with B‐O‐Cu5. Interestingly, when we employed the B site on B‐O‐Cu2 for additional ^*^CO adsorption, it exhibited lower energy barriers (^*^COH, TS, and coupling) due to stronger ^*^CO_L_ adsorption on B. Experimental in situ Raman results also indicated enhanced ^*^CO adsorption. Furthermore, the stronger B─CO bonding raised the difficulty of dehydroxylation of ^*^COH. This resulted in an alternative pathway involving H addition, followed by hydrogenolysis to produce ^*^HCCOH, which had lower energy compared to ^*^HCCHO by 0.33 eV. This suggests the advantage of B as an adsorbate site for ^*^CO in facilitating the differentiation of C_2+_ products, the corresponding divergent charge differential distributions of ^*^CCO and ^*^HOCCOH are also shown in Figure 6b.

We utilized constant electrode potential (CEP) density functional theory calculations to investigate the C–C coupling process during CO_2_ electrochemical reduction over various catalysts. The CEP model was used to calculate the solvation and applied potential effects on C–C coupling, which plays a key role in the formation of C_2_ products in CO_2_RR. The results were compared with those obtained from the computational hydrogen electrode (CHE) model. To determine the potential effects, CEP calculations were completed at U = −0.5 and −1.0V vs the SHE. Figure S41 (Supporting Information) shows that the kinetic barrier for the C–C coupling step decreases as the voltage becomes more negative, according to the CEP calculations. The CEP model predicts energy barriers of 0.36 eV and 0.27 eV for U = −0.5V and −1.0 V vs the SHE in TS (Cu‐L1), respectively, which are slightly lower than the 0.43 eV predicted by the CHE model. The TS Gibbs free energy of the C‐C coupling process at the B site on Cu‐L0 varies very little, indicating relative stability with voltage. Additionally, the CEP model predicts that the kinetic barrier for the C–C coupling step is only slightly lower than that predicted by the CHE model for various catalysts. Although the use of the CEP model may decrease the free energy barrier, the energy barrier values for C–C coupling show good consistency between the CHE and CEP models in our calculations for all catalysts. This means that both the CHE and CEP models can achieve similar results and are suitable for CO_2_RR over these catalysts.

Next, we needed to continue exploring the intermediate pathways for C_2+_ product differentiation on three catalytic configurations. Specifically, we focused on the corresponding reaction energies for the generation of ethylene and ethanol products after initiation with ^*^HCCOH and ^*^HCCHO (as shown in Figure 6e). In the Cu(111) and Cu‐L1 configurations with ^*^HCCHO as the promoter at the Cu site, ^*^H tends to be added to C due to the relatively weak Cu─CO bond energy. This leads to an increase in the energy of ^*^HCCHOH on O, and an increase in the free energy of the ethylene pathway by 0.15 eV compared to the ethanol pathway. This suggests that C_2+_ product partitioning at the Cu site facilitates the protonation of C and subsequent retention of −OH in Cu─C─O, which is essential for subsequent ethanol formation. In the Cu‐L1 configuration, the presence of interlayer B elements leads to lattice expansion and the formation of electronically delocalized domains within the Cu crystalline phase, as observed by XRD and XPS experiments. Calculations show lower interstitial adsorption energies, indicating higher stability. In the presence of B, the free energy change of the C_2+_ product is 0.07 eV lower than that of Cu(111), indicating that B doping enhances the catalytic activity of CO_2_RR. Notably, the free energy change of the ^*^HCCOH pathway is usually lower than that of ^*^HCCHO, and the subsequent hydrogenation and dehydration processes become easier due to the addition of H on O, resulting in a continuous decrease in the overall stepwise trend. Since H─O─ leaves easily with O, the ─OH group cannot be favorably retained, ultimately leading to ethylene production.^[^ ^20c^ ^]^ The optimal ethylenic ethanol product differentiation routes and atom migration steps are shown by us in Figure 6f. Throughout the entire reaction pathway, when Cu or B‐doped Cu is used, the final products predominantly consist of ethanol. The introduction of B improves the adsorption of ^*^CHO on Cu sites, confirming the ethanol production advantage of the B‐O‐Cu5 sample. Interestingly, when B acts as an additional adsorption site for ^*^CO, the strong adsorption of ^*^CO truncates the ethanol pathway, and the ^*^COH coupling pathway promotes the formation of ethylene. Moreover, the low ^*^CO adsorption and coupling pathway also enhance the activity for generating C_2+_ products.

## 3 Conclusion

In this research, we investigated the efficient conversion of CO_2_ into C_2+_ products using B‐O‐Cux@Ni‐SACy tandem catalysts. By tuning the B doping levels and the ratio of Ni‐SAC, we achieved high selectivity for ethylene and ethanol, with faraday efficiencies of 72% and 48%, respectively. Our study revealed the importance of B doping in enhancing the adsorption of ^*^CO and influencing the pathways for C_2+_ product differentiation. The tandem catalyst approach, incorporating an additional source of ^*^CO generation, provided valuable insights into improving CO_2_ electrocatalysis and holds promise for the sustainable production of C_2+_ products from carbon dioxide.

## 4 Experimental Section

### Method—Synthesis of B‐O‐Cux

B‐O‐Cux samples were prepared by a simple one‐step method using copper chloride (CuCl_2_) and sodium borohydride (NaBH_4_) as precursors. Due to the low solubility of boron in copper, CuCl_2_ was immediately added to a highly concentrated solution of sodium borohydride to maximize the alloying of boron with copper. First, a quantitative amount of CuCl_2_ and NaBH_4_ solid powder was dissolved in chilled water (≈0 °C). Next, 2 mL of a certain concentration of CuCl_2_ solution was quickly injected into a rapid injection of NaBH_4_ (5 m, 2 mL) solution until no bubbles were produced. The obtained precipitate was subsequently washed three times with 150 mL of water (50 ml each time) and once with 50 mL of acetone to completely remove unreacted precursors and other possible by‐products. Then, the powder was immediately dried under a vacuum overnight. The amounts of CuCl_2_ for the different B‐doped Cu samples were as follows: 400 mg for B‐O‐Cu1, 300 mg for B‐O‐Cu2, 200 mg for B‐O‐Cu3, 100 mg for B‐O‐Cu4, 50 mg for B‐O‐Cu5.

### Method—Synthesis of Cu_H_

Cu_H_ samples were prepared by a simple one‐step method using copper chloride (CuCl_2_) and hydrazine hydrate (N_2_H_4_) as precursors. First, N_2_H_4_ (1mL) was dissolved in 2mL chilled water (≈0 °C). Next, 2 mL of a certain concentration of CuCl_2_ (300mg) solution was quickly injected. The obtained precipitate was subsequently washed three times with 150 mL of water (50 mL each time) and once with 50 mL of acetone to completely remove unreacted precursors and other possible by‐products. Then, the powder was immediately dried under a vacuum overnight. The synthesis method of Cu_H_‐1 is similar to the above‐described procedure, conducted at 4 °C, with the addition of 1.5 mL of N_2_H_4_·H_2_O solution.

### Method—Synthesis of Ni‐SAC

According to the previous report, Ni‐SAC was initially synthesized by preparing a “precisely pre‐buried” Ni‐salen crystalline polymer, followed by high‐temperature pyrolysis to obtain Ni‐SAC. In the synthesis process, 1,2,4,5‐benzenetetramine hydrochloride (59.0 mg, 0.208 mmol) was dispersed in a 50 mL hydrothermal kettle containing a mixture of 1,3,5‐trimethylbenzene and ethanol solvent (8.4 mL) in a 2:1 ratio. After adding triethylamine (120 µL, 0.832 mmol), the mixture was subjected to ultrasonic mixing for 5 min. Subsequently, Ni(OAc)_2_·4H_2_O (102.88 mg, 0.416 mmol), 3,3'‐dialdehyde‐4,4'‐dihydroxybiphenyl (100 mg, 0.416 mmol), and aqueous acetic acid solution (1 mL, 6.0 m) were added to the hydrothermal kettle, and the resulting mixture was sonicated for an additional 5 min. Finally, the mixture was heated to 80 °C for 5 days to synthesize a reddish–brown solid. The solid was filtered, washed with ultrapure water, anhydrous ethanol, tetrahydrofuran, and acetone, and then dried under vacuum at 160 °C for 12 h to obtain a reddish–brown powder.

To perform high‐temperature pyrolysis, 8 mmol of sodium citrate was pyrolyzed in a tube furnace under an argon atmosphere at 800 °C for 1 h. The resulting black solid product was washed with a 0.5 m HCl solution and ultrapure water to remove inorganic impurities. After drying at 70 °C, PC scaffolds were obtained. Then, PC (10 mg), dicyandiamide (125 mg), and Ni‐salen crystalline polymer (50 mg) were added to a mortar and ground for 30 min. The resulting powder was placed in a tube furnace and heated to 650 °C under an argon flow rate of 100 mL min^−1^. After 200 min of pyrolysis, Ni‐SAC was obtained.

### Characterization

To ensure sample stability, all specimens were subjected to testing characterization after a minimum of 2 h of quiescence in ambient air. The powder XRD patterns were collected on a SmartLab 9 kW instrument using Cu Kα radiation. The SEM images were obtained using a field emission environmental scanning electron microscope (Quanta 200FEG). The TEM images were obtained on a JEM–2100 microscope at an acceleration voltage of 200 kV. The HAADF–STEM observations were performed using an FEI Themis Z instrument. A PerkinElmer NexION 300X ICP‐MS spectrometer was used to quantify metal contents. The XPS profiles of the studied materials were acquired using a Thermo Scientific K‐Alpha instrument equipped with an Al‐Kα radiation source. The Raman spectra were recorded on Beijing Zolix Instruments, Inc. equipped with two‐edge Rayleigh filters and an interference filter for plasma line removal. The synchrotron radiation experiment was performed at the easyXAFS300 of Quantum Design company, American, in which, the measurable energy range: 5–18KeV, and the test mode is transmission mode.

### Characterization—ICP‐MS Test

10 mg sample was completely dissolved in 100 mL of dilute HNO_3_ solution (5 v.%) and subjected to ultrasonication for 30 min, trace insoluble matter was removed from 5 mL solution through a water‐based membrane for ICP‐MS test.

### Characterization—Time‐Dependent ICP‐MS Test

1 mg sample was completely dissolved in 50 mL of dilute HNO_3_ solution (5 mM) and subjected to ultrasonication for 30 min prior to conducting ICP‐MS testing. At time intervals of 10 seconds, 2 min, 5 min, 10 min, and 30 min, 5 mL solution was extracted for testing.

### Characterization—Electrochemical In Situ ATR‐FTIR Test

Electrochemical in situ Attenuated Total Reflection Flourier Transformed Infrared Spectroscopy (ATR‐FTIR) was employed to trace the signals of the intermediates using a Nicolet Nexus 670 Spectroscopy equipped with a liquid nitrogen‐cooled mercury‐cadmium‐telluride (MCT) detector. An ECIR‐II cell equipped with a Pike Veemax III ATR in a three‐electrode system was provided by Shanghai Linglu Instrument& Equipment Co. To improve the signal intensity, the monocrystal silicon was initially coated with a layer of Au using the chemical plating method. Then, 20 µL catalyst ink was dropped on the surface of the Au film and served as the working electrode. Platinum sheet and Ag/AgCl electrode were used as counter electrodes and reference electrodes, respectively. Before the test, the CO_2_ feeding gas was purged into the electrolyte and continuously bubbled during the measurement. The dropped catalysts were electroreduced at −0.4 V vs. RHE for 30 min to be active for the test. The potential‐dependent in situ ATR‐FTIR tests were carried out with LSV test from −0.4 to −1.4 V (vs. RHE) with a scan rate of 5 mV s^−1^.

### Characterization—Electrochemical In Situ Raman Test

The in situ Raman measurement was carried out on a Raman spectroscopy equipped with a 532 nm laser (RTS2‐301‐SMS, Zolix Instruments, Inc.). The measurements were performed in an electrochemical cell with a Pt wire and an Ag/AgCl electrode as the counter and reference electrode, respectively. For Potential‐resolved operando Raman spectroscopy, the collection time was 20 min, and the exposure and accumulation times were 10 s.

### Characterization—Electrochemical In Situ XAFS Test

X‐ray absorption fine structure (XAFS) spectroscopy was carried out using the *RapidXAFS* 2M (Anhui Absorption Spectroscopy Analysis Instrument Co., Ltd.) by transmission(or fluorescence) mode at 20 kV and 20 mA, and the Si (553) spherically bent crystal analyzer with a radius of curvature of 500 mm was used for Cu.

### Electrochemical Measurements

All the electrochemical CO_2_/CO reduction measurements were conducted in a flow cell controlled with an electrochemical workstation (CHI760E). The flow cell consists of three independent chambers: catholyte (gas diffusion electrode, SGL Sigracet 28 BC), gas, and anolyte (Platinum flakes, 1 mm in thickness), as shown in Figure S8 (Supporting Information). The Ag/AgCl reference electrode was placed into the catholyte chamber through a drilled top hole. A proton exchange membrane (N117) was employed to separate the anolyte and catholyte chambers. The working electrode was prepared by spray‐coating a pre‐made catalyst ink via an airbrush on the gas diffusion electrode with a size of 2 × 0.5 cm^2^. The catalyst loading is 1mg cm^−2^ to ensure similar particle density. 0.5 m aqueous KHCO_3_ solution was circulated into both the anode and cathode chambers at a constant flow rate of 10 ml min^−1^ with the assistance of a dual‐channel peristaltic pump. A high‐purity CO_2_/CO gas flow of 10 cm^3^ min^−1^ was supplied to the gas chamber controlled by a digital mass flow controller (Horiba). The gas products were quantitatively analyzed using gas chromatography equipped with both flame ionization and thermal conductivity detectors (Fuli GC9790 Plus). The liquid products were collected after at least 3 h of electrolysis and quantitatively analyzed using ^1^H NMR spectroscopy with H_2_O suppression. 300 µL electrolyte mixed with 10 µL dimethyl sulfoxide (20 mM) and 200 µL D_2_O was used as the internal standard. All the potentials were converted to RHE, according to the equation:

(1) $$E\left(\mathrm{vs}.\;\mathrm{RHE}\right)=E\left(\mathrm{vs}.\;\mathrm{Ag}/\mathrm{AgCl}\right)+0.059\mathrm{pH}+0.198$$

Electrode intrinsic properties of the analyzed materials were evaluated on a three‐electrode system using an Ag/AgCl electrode as the reference electrode, graphite as the counter electrode, and a KHCO_3_ solution (0.5 M) as the electrolyte. The flow rate of CO_2_/CO was maintained at 10 mL min^−1^ by a mass flowmeter during electrochemical measurements. The LSV measurements were conducted at a scan rate of 5 mV s^−1^ in a KHCO_3_ solution (0.5 m). The ECSAs of the prepared catalysts were compared by measuring their double‐layer capacitances calculated from the corresponding cyclic voltammetry curves in the potential window from −0.05 to 0.05 V vs. RHE. The EIS experiments for CO_2_/CO reduction at the open‐circuit potential were performed at a small (5 mV) AC voltage in a frequency range from 10 MHz to 100 kHz.

The FE of the gas products was calculated as follows:

(2) $$\begin{array}{ccc} \mathrm{FE}=\frac{Q_{\mathrm{product}}}{Q_{\mathrm{total}}}\times 100\% & = & \frac{\left(\frac{v}{60s\min^{-1}}\right)\times \left(\frac{y}{24000\;cm^{3}\;\mathrm{mo}l^{-1}}\right)\times N\times F}{j}\\ & & \times \;\;100\% \end{array}$$

where v is the CO_2_/CO flow rate (10 mL min^−1^); y is the concentration of gas in a 1 mL quantitative loop (ppm); N is the electron transfer number; F is the Faraday constant (96485 C mol^−1^); and j is the total current density (A cm^−2^).

The FE of the liquid products: *Q_product_* and *Q_total_* were obtained using the following equations:

(3) $$Q_{product}=Z_{product}\times F\times N_{product}$$

(4) $$Q_{\mathrm{total}}=I\times t$$

Therefore, *FE*_liquid_ can be written as

(5) $$FE_{\mathrm{liquid}}=\frac{Z_{product}\times F\times N_{product}}{I\times t}\;$$

*Z_product_* is the number of electrons exchanged for the product formation; *N_product_* is obtained by quantifying the DMSO solution in the H‐NMR spectrum as an internal reference calibration.

### Electrochemical measurements—Electrochemical Active Surface Area (ECSA) Calculation

The ECSAs of catalysts were calculated based on their electrical double layer capacitor (Cdl), which were obtained from CV plots in a narrow non‐Faradaic potential window from 0.05 to −0.05 V (vs. RHE) in H‐cell. The measured capacitive current densities at 0 V were plotted as a function of scan rate and the slope of the linear fit was calculated as Cdl. The specific capacitance was found to be 60 µF cm^−2^, and the ECSA of the catalyst is calculated from the following equation:

(6) $$\mathrm{ECSA}=C_{\mathrm{dl}}/\left(60\mu F\;cm^{-2}\right)cm^{2}$$

The intrinsic activity was revealed by normalizing the current to the ECSA to exclude the effect of surface area on catalytic performance.

### DFT Calculations—First‐Principle Computational Procedure

The plane‐wave Vienna Ab‐initio Simulation Package (VASP) was applied in all first‐principles calculations using projector augmented‐wave pseudopotentials with the GGA‐type PBE functional.^[^ ^25^ ^]^ A model with periodic boundary conditions was used, and the plane wave energy cutoff was set to 450 eV. The reciprocal space for all calculation systems was a Monkhorst–Pack k‐point grid with dimensions of 4 × 4 × 6. To prevent interactions between replicas along the z‐direction, vacuum spacing of at least 20 Å was used between the adjacent images.

The adsorption energy was calculated by the following formula:

(7) $$E_{a}=E_{\mathrm{final}}-E_{\mathrm{substrate}}-E_{\mathrm{adsorbate}}$$

where *E_a_*, *E_final_*, *E_substrate_*, and *E_adsorbate_* are the adsorption energy of the adsorbate on the substrate, total energy of the adsorbate on the substrate, total energy of the substrate, and total energy of the adsorbate, respectively.

The free energy of this reaction was computed via the following equation: *∆G* = *E_a_* + *∆Z_PE_* − *T∆S*, where *∆G*, *∆Z_PE_*, and *∆S* represent Gibbs free energy, zero‐point energy, and entropy, respectively.

### DFT Calculations—The Constant Electrode Potential (CEP) Model DFT Calculation

The solvation effect was treated as a polarizable continuum based on the linearized Poisson−Boltzmann model, implemented in VASPsol,^[^ ^26^ ^]^ with the Debye length of 3 A corresponding to the concentration of 1 m electrolyte. The Debye length of 3 A was selected based on previous experimental and computational studies.^[^ ^27^ ^]^ The reaction‐free energies at different applied potentials were calculated using the CEP model. In the CEP model, a constant potential (*U*) was achieved by varying the number of electrons to adjust the Fermi level of the system with respect to the standard hydrogen electrode (SHE). The relationship between the applied potential, Fermi level, and SHE is expressed as:

(8) $$U=\left(-E_{f}-\Phi_{SHE}\right)/\left|e\right|$$

A value of 4.3 eV, which was suggested by Anderson and coworkers in their theoretical study,^3^ was used for ΦSHE. In the CEP model, the calculated free energy needs to be corrected for a different number of electrons in the charged and neutral systems under the applied potential. This correction (*G*(*U*)) can be determined by:

(9) $$G\left(U\right)=G\left(N_{e,U}-N_{e,0}\right)\mu_{e}\left(U\right)$$

where *N*_e,0_ and *N*_e,U_ are the number of electrons of the neutral and corresponding charged system, respectively and *µ*_e_(*U*) is the chemical potential of the electron under the applied potential, i.e., the Fermi energy of the system. The Δ*G*(*U*)s were corrected for the finite cell volume by Gauthier et al.^[^ ^28^ ^]^

## Conflict of Interest

The authors declare no conflict of interest.

## Supporting information



Supporting Information

## Data Availability

The data that support the findings of this study are available from the corresponding author upon reasonable request.
