# Supplementary material for: Enhancing CO2 Electroreduction Precision to Ethylene and Ethanol: The Role of Additional Boron Catalytic Sites in Cu‐Based Tandem Catalysts
Source: Adv Sci (Weinh). 2024 Oct 21;11(46):2410118. doi: 10.1002/advs.202410118 (PMC11633483; doi:10.1002/advs.202410118)
Supplement: Supplementary file 1 — Supporting Information [file ADVS-11-2410118-s001.docx]

Supporting Information

Enhancing CO_2_ Electroreduction Precision to Ethylene and Ethanol: The Role of Additional Boron Catalytic Sites in Cu-Based Tandem Catalysts

Fuqing Yu, Minxing Shu, Guangyao Zhang, Qiming Yu, Hongming Wang*

**Chemicals**

Cupric chloride (CuCl_2_·H_2_O, purity>99%), Sodium borohydride (NaBH_4_, >99%), Methanol (CH_3_OH), ethanol, potassium bicarbonate (KHCO_3_, ≥99.5%), Nickel acetate tetrahydrate (Ni(OAc)_2_·4H_2_O, ≥99.5%), triethylamine, sodium citrate (>99%), Hydrochloric acid (HCl, vol%>37%) were purchased from China National Medicines Corporation Ltd. 1,2,4,5-benzenetetramine hydrochloride, 1,3,5-trimethylbenzene were purchased from Shanghai Aladdin Bio-Chem Technology Co., LTD. All chemicals were used without further purification.

All materials were used directly with no further purification. Milli-Q (DI) water (>18.0 MΩ) was purified with a Sartorius arium mini ultrapure water system.


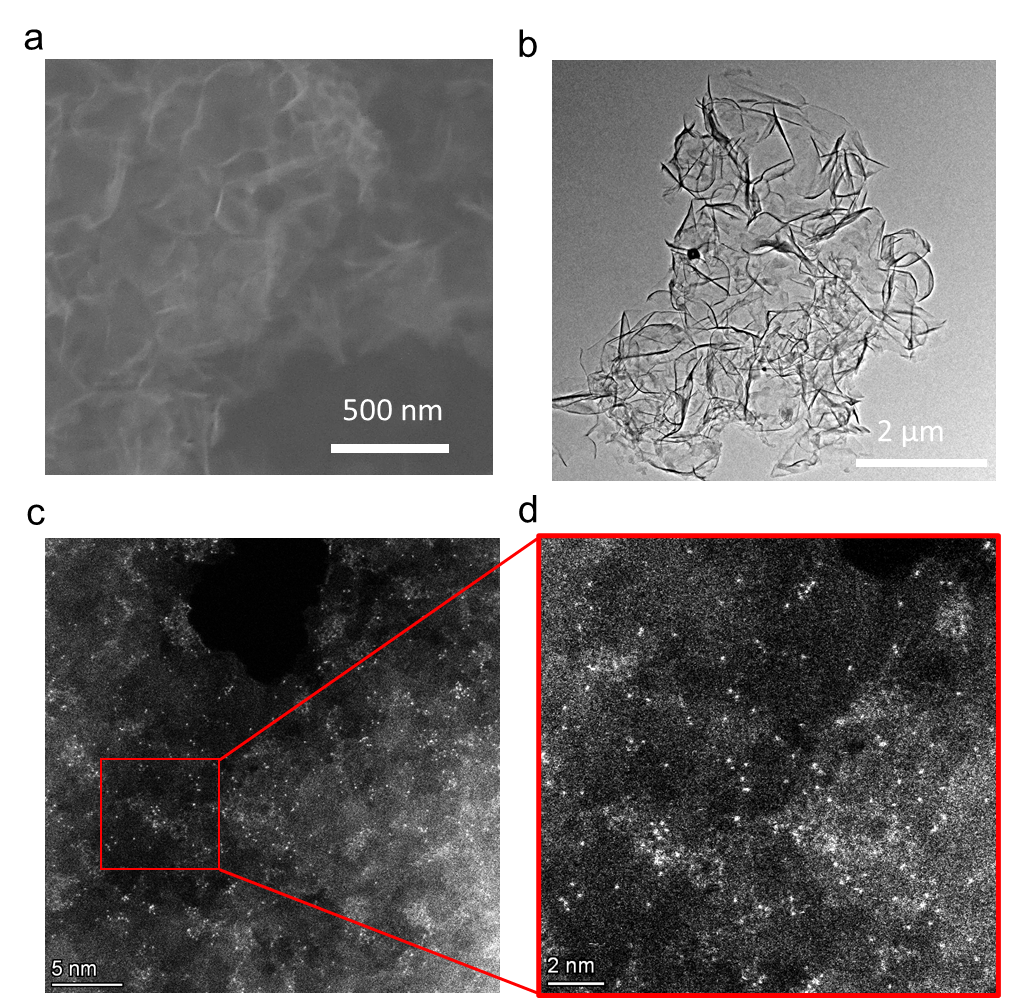


Figure S1 . a) SEM , b)TEM and c) HAADF-TEM images of Ni-SAC. d) Magnified HAADF-TEM images of Ni atomic dispersion.


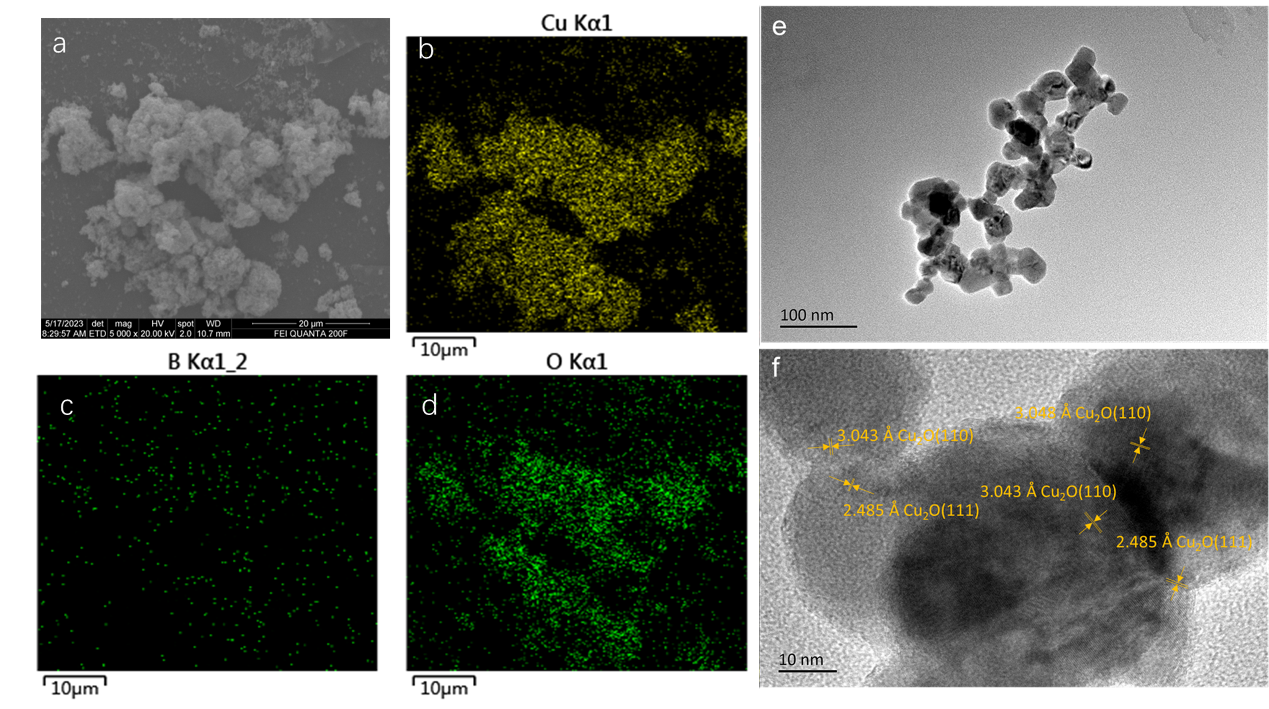


Figure S2. a) SEM images and Mapping of b) O c) B d) Cu elemental surface distributions of B-O-Cu2. e) TEM and HRTEM image of B-O-Cu2.


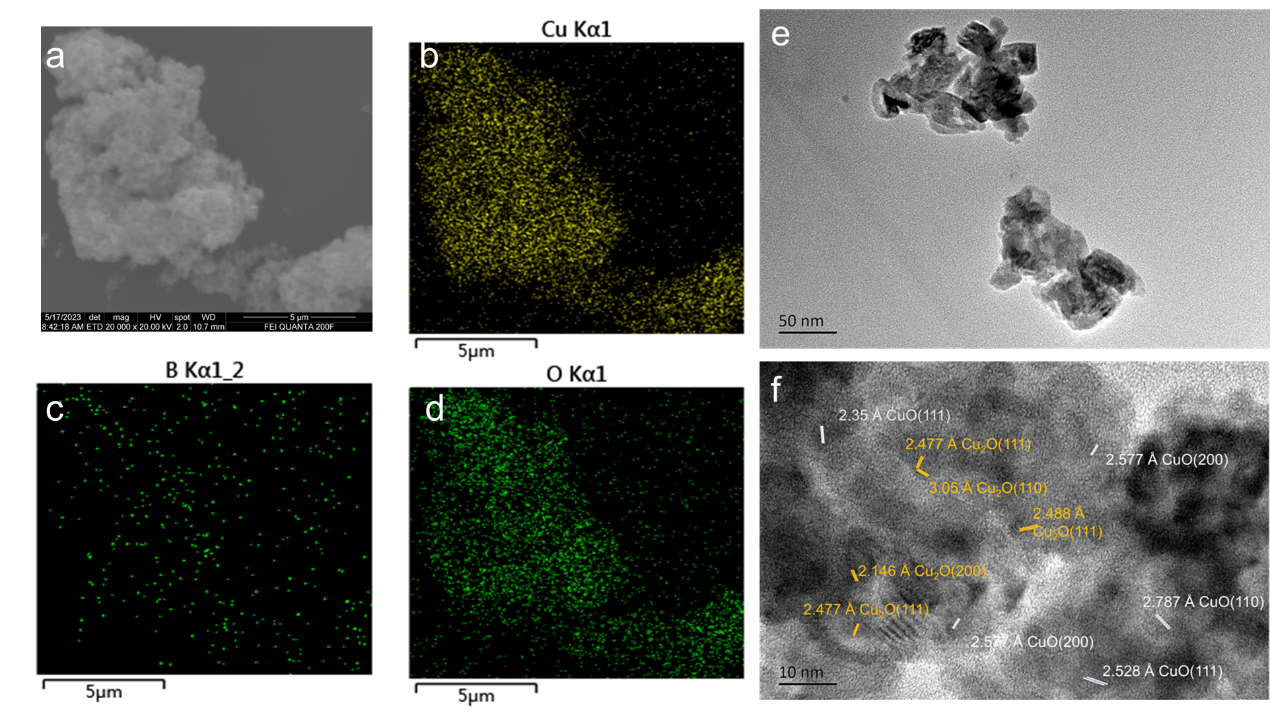


Figure S3. a) SEM images and Mapping of b) O c) B d) Cu elemental surface distributions of B-O-Cu5. e) TEM and HRTEM image of B-O-Cu5.


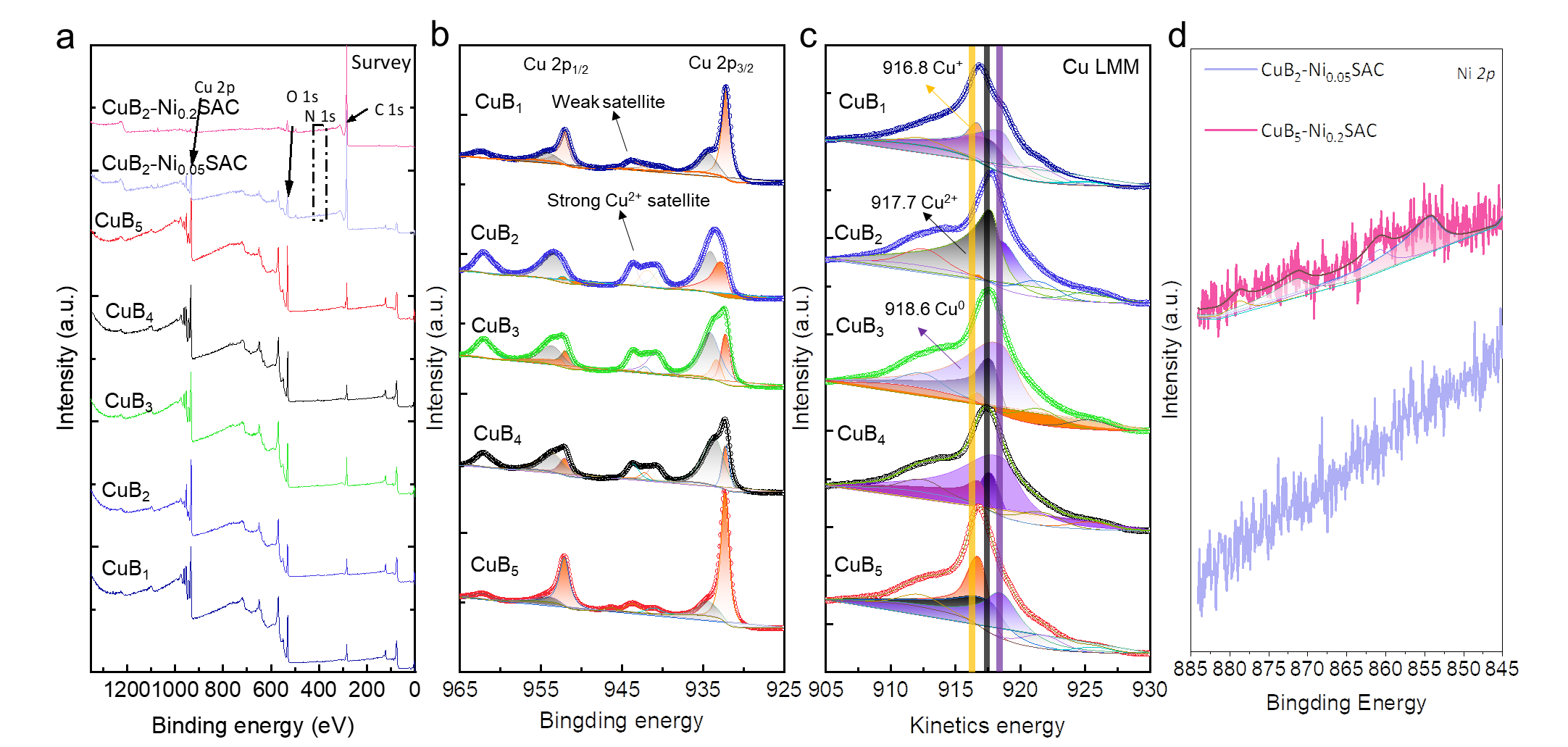


Figure S4 a) Survey, b) Cu 2p c) Cu LMM d) Ni 2p XPS spectra of the B-O-Cux@Ni-SACy.


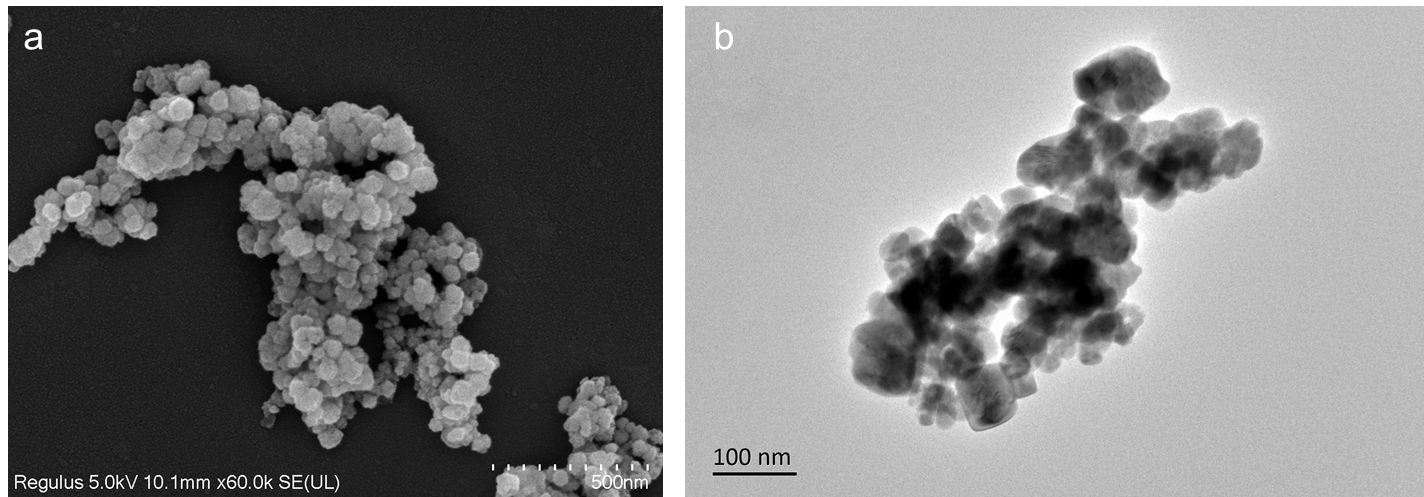


Figure S5. a) SEM and b) TEM images of Cu_H._

**
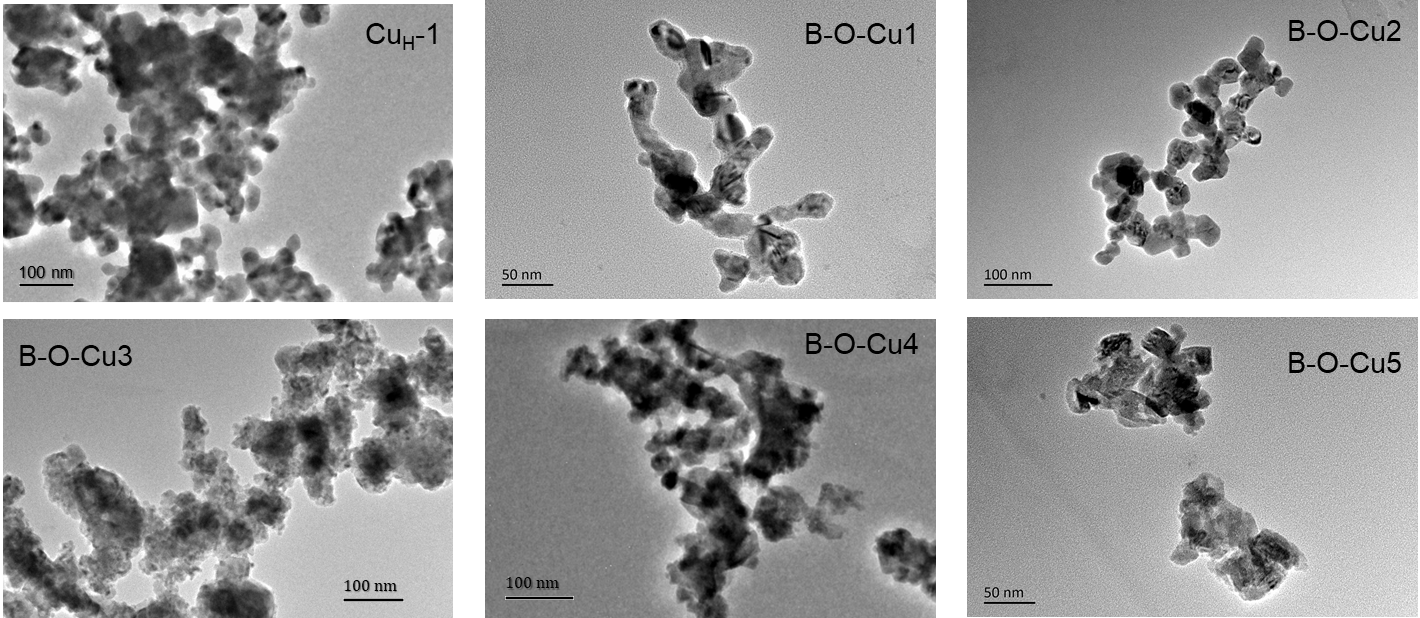
**

Figure S6. TEM Images of B-O-Cux Samples with Increasing B Content from B-Free to B-Containing.


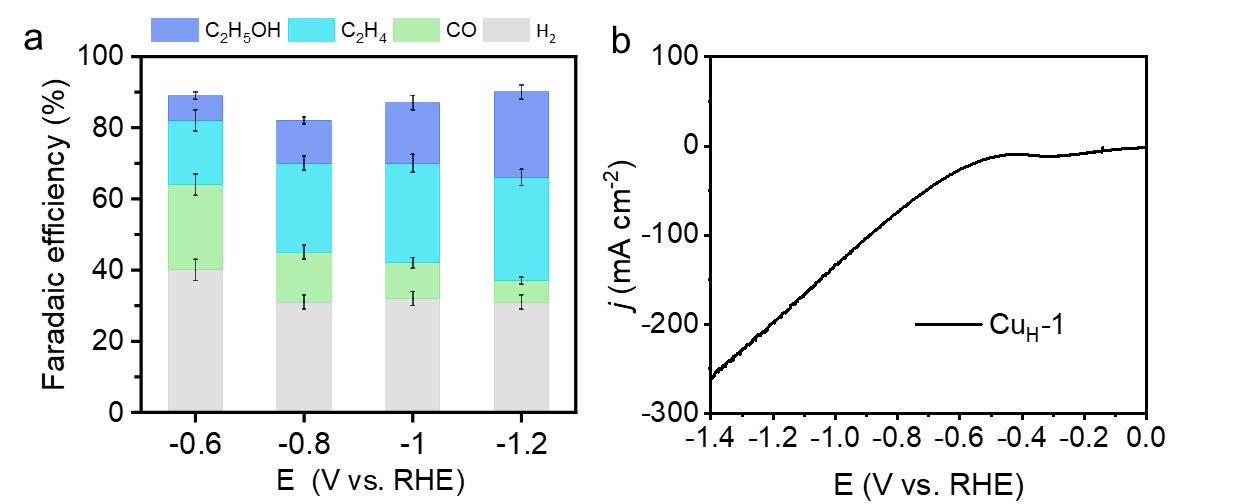
 Figure S7. a) the FE performance at -0.6 to -1.2 V vs. RHE of Cu_H_-1 in 0.5 M CO_2_- saturated KHCO_3,_ and corresponding b) Linear voltametric curves.

**
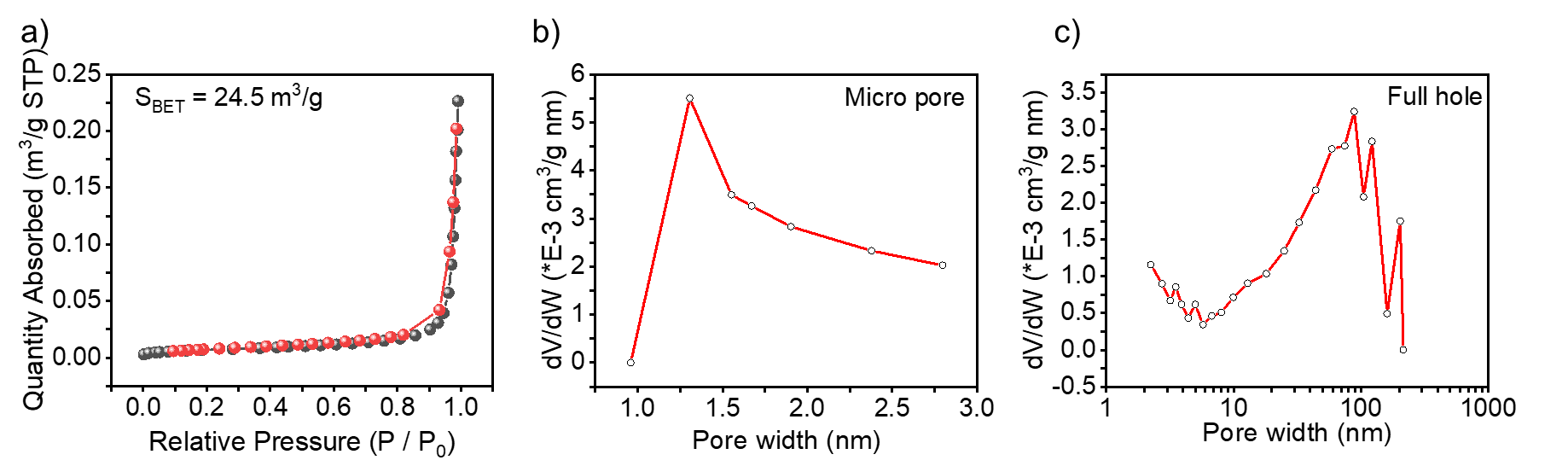
**

Figure S8. a) Adsorption and desorption isotherm and specific surface area measurements of B-O-Cu2 sample. b,c ) Pore size distribution of B-O-Cu2 micropores and whole pores, respectively.


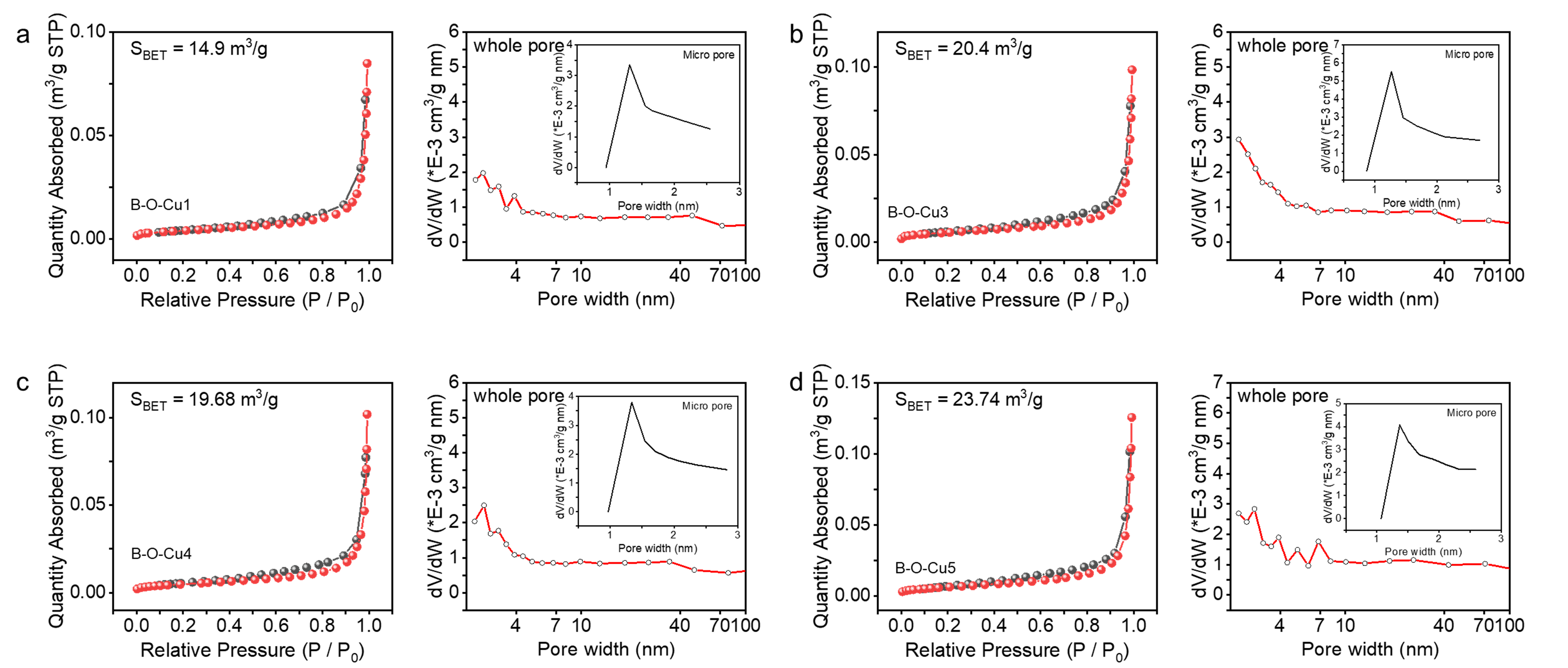


Figure S9. Adsorption and desorption isotherm, specific surface area and Pore size distribution measurements of a) B-O-Cu1, b) B-O-Cu3, c) B-O-Cu4 and d)B-O-Cu5,sample, respectively.


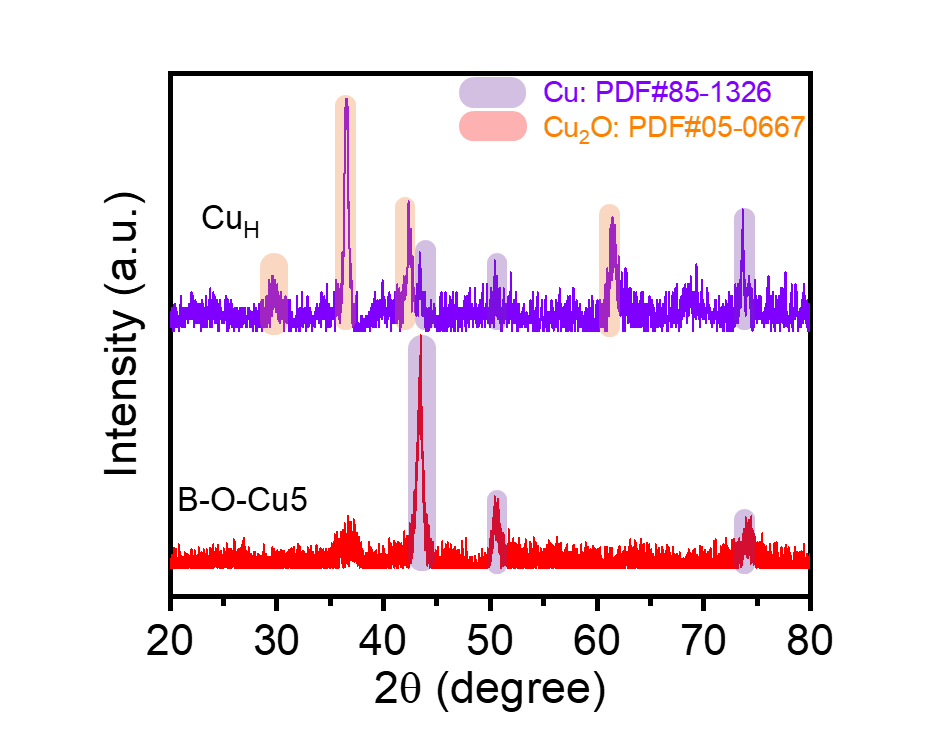


Figure S10.XRD images of Cu_H_ and B-O-Cu5_._


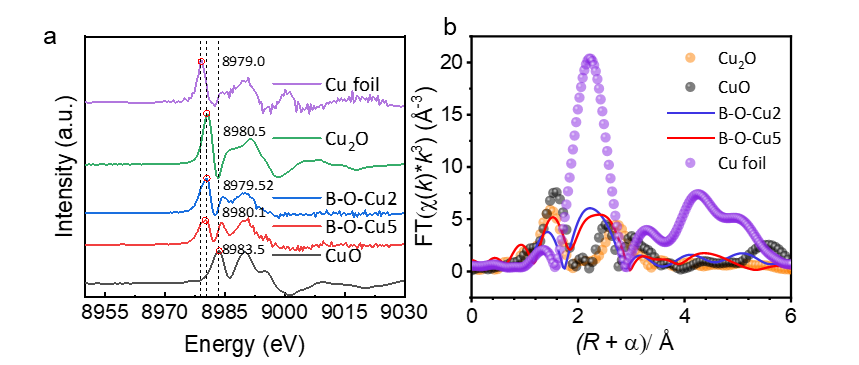


Figure S11. a) The near-edge first-order derivative diagram of Cu K-edge Survey of B-O-Cux, b) Cu K-edge EXAFS curve fit for B-O-Cu2, B-O-Cu5 and standard sample shown in R-space. The data are k^3^-weighted and not phase-corrected.


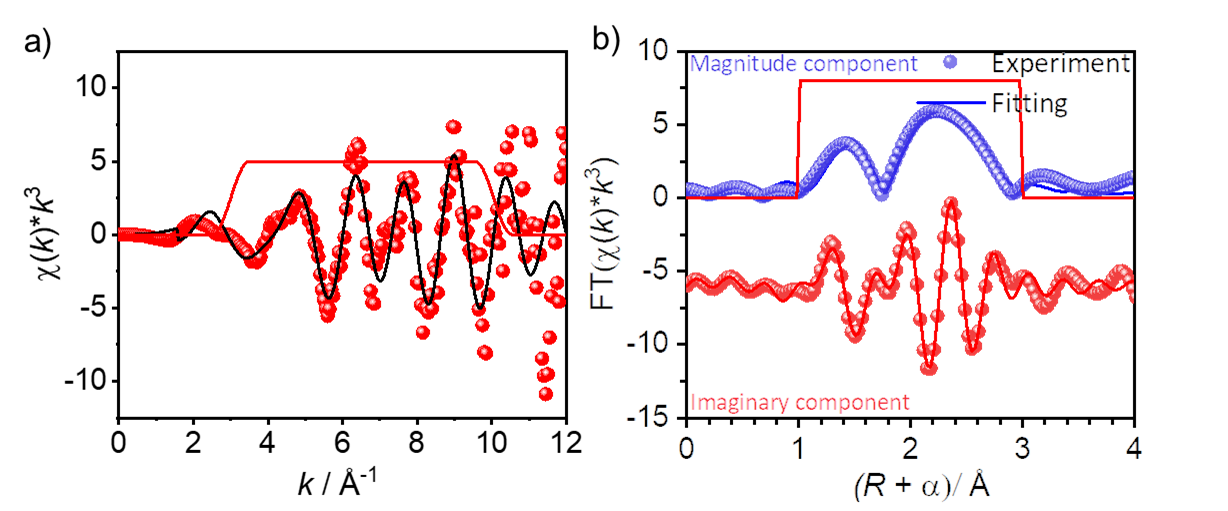


Figure S12. a) Cu K-edge EXAFS (points) and the curvefit (line) for B-O-Cu2 shown in k^3^weighted *k*-space. b) Cu K-edge EXAFS (points) and curvefit (line) for B-O-Cu2 shown in R-space (FT magnitude and imaginary component). The data are k^3^-weighted and not phase-corrected.


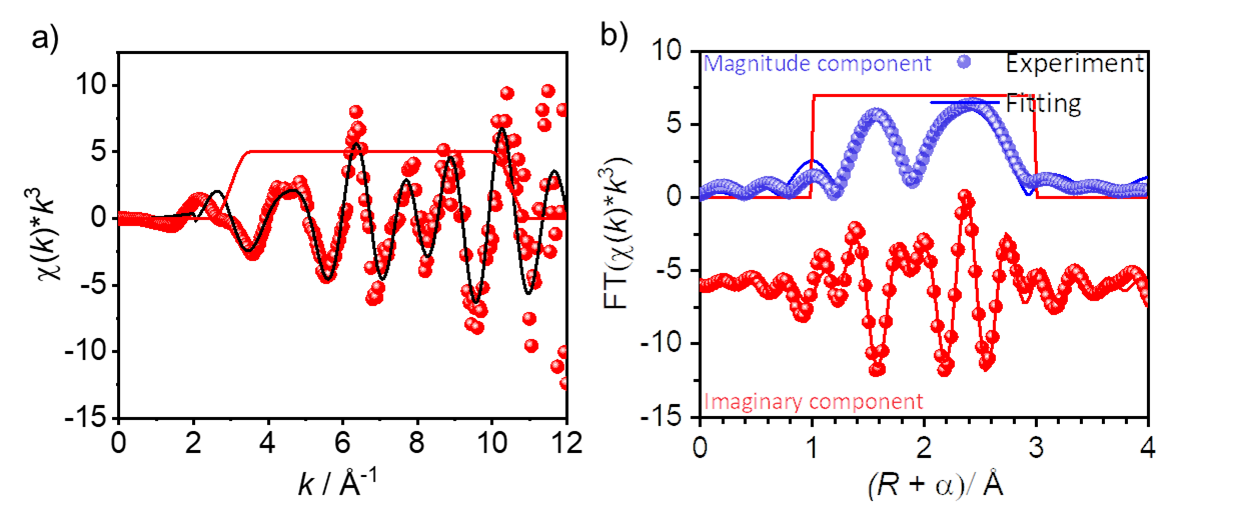


Figure S13. a) Cu K-edge EXAFS (points) and the curvefit (line) for B-O-Cu5 shown in k^3^weighted *k*-space. b) Cu K-edge EXAFS (points) and curvefit (line) for B-O-Cu5 shown in R-space (FT magnitude and imaginary component). The data are k^3^-weighted and not phase-corrected.

**
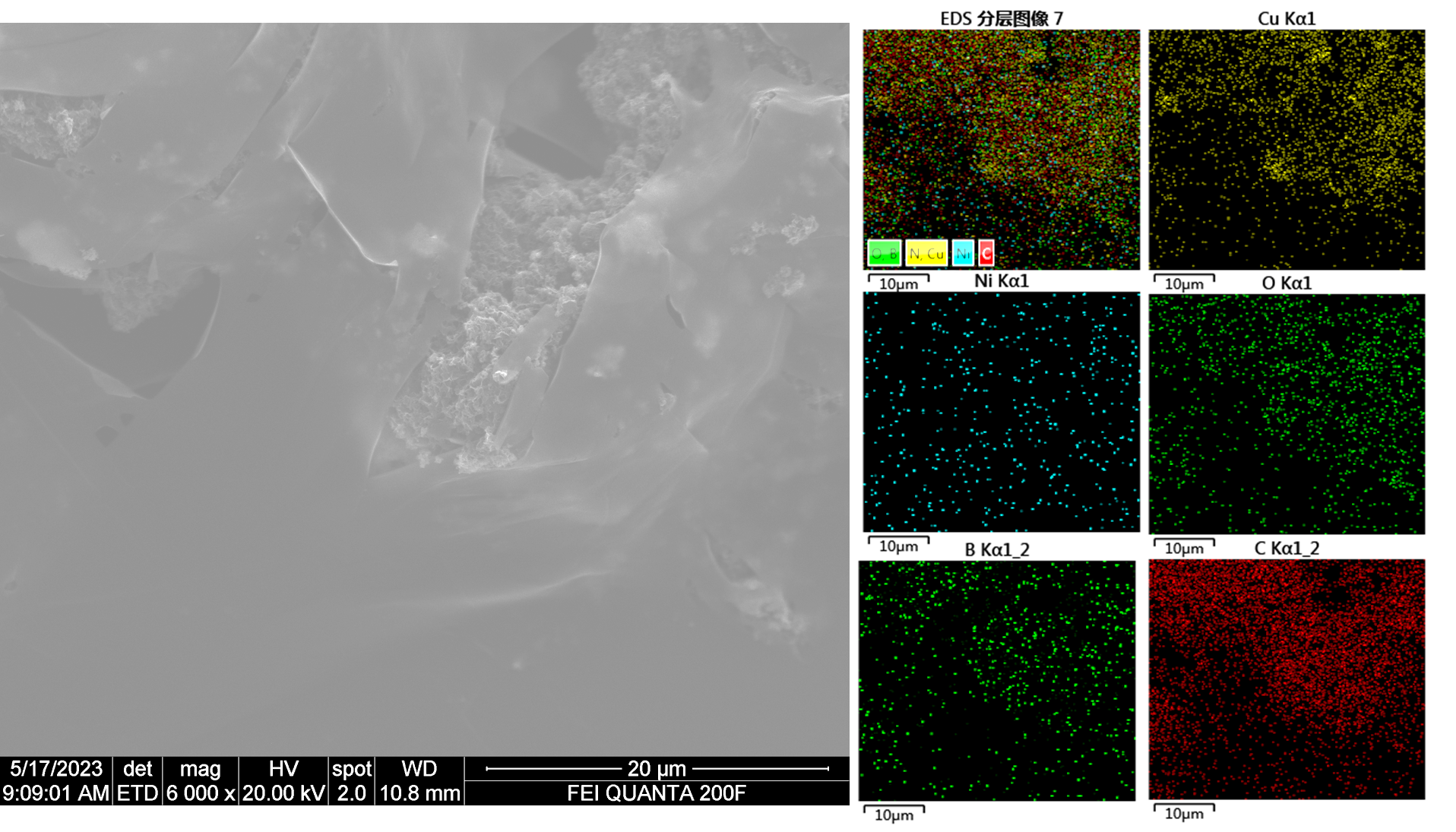
**

Figure S14. SEM images and Mapping of B, O, Cu, Ni, and C elemental surface distributions of B-O-Cu2@Ni-SAC0.05.


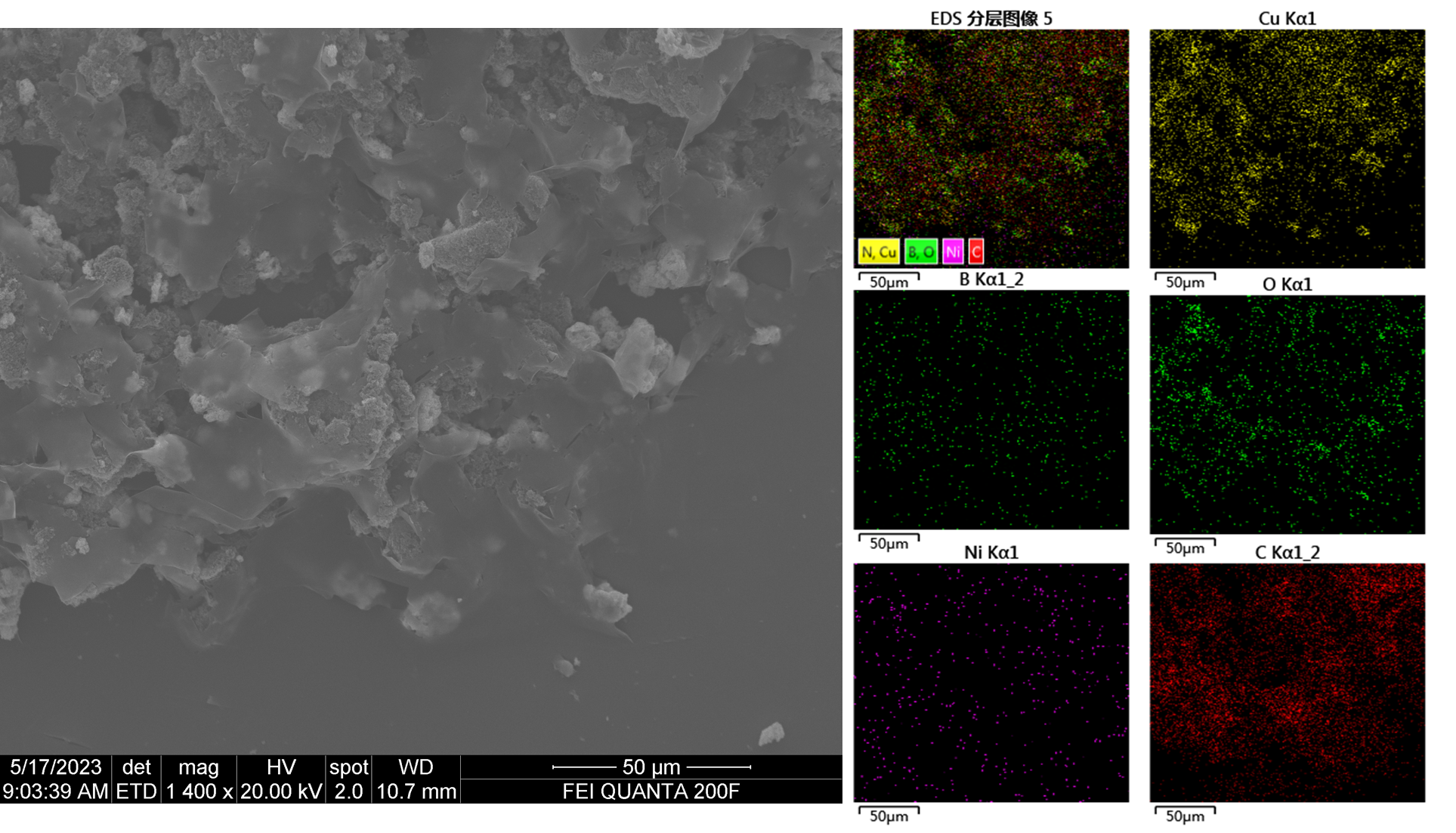


Figure S15. SEM images and Mapping of B, O, Cu, Ni, and C elemental surface distributions of B-O-Cu5@Ni-SAC0.2.


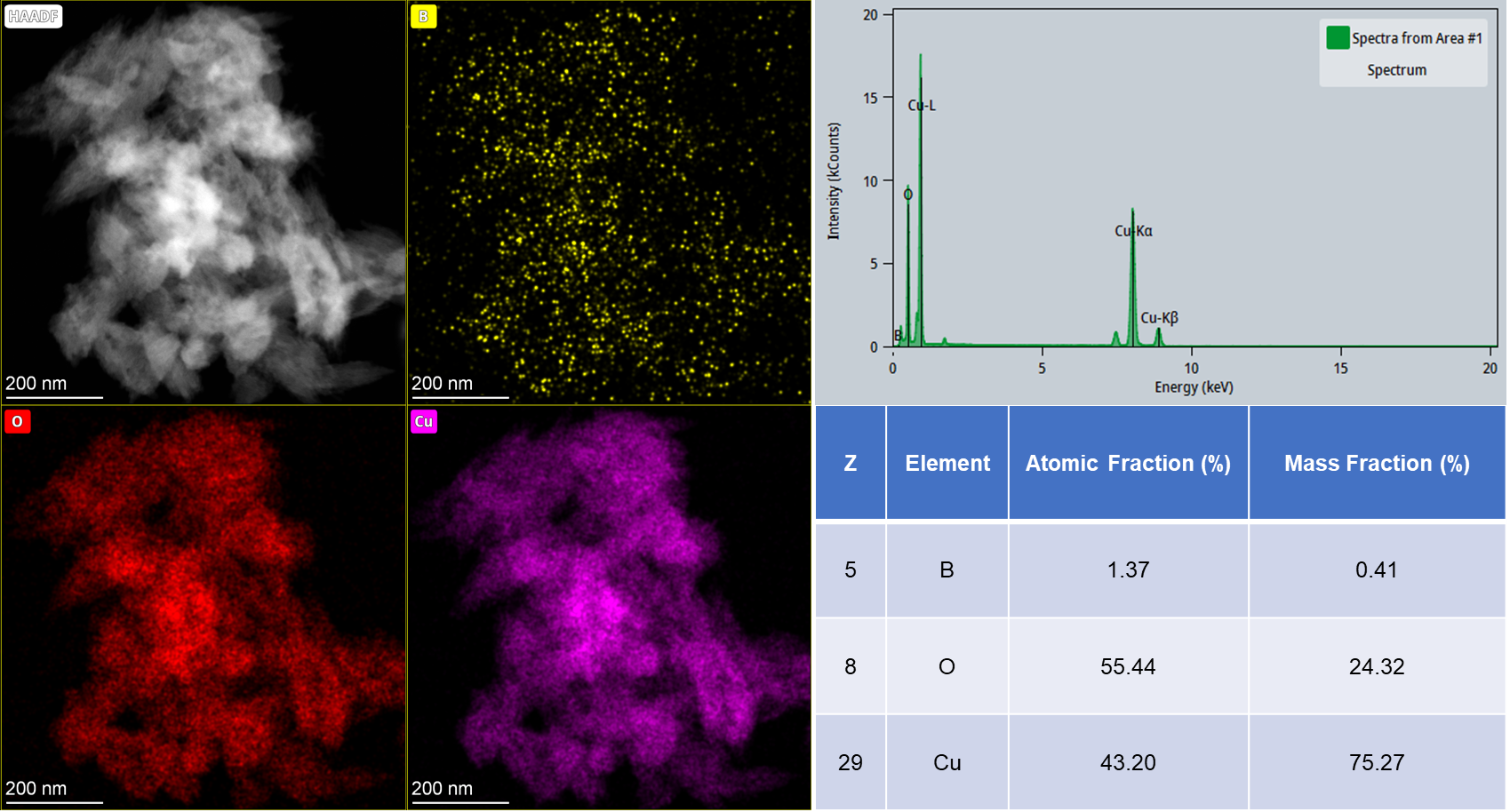


Figure S16. The elemental mapping and content ratios of the high-resolution TEM of the B-O-Cu2 samples.


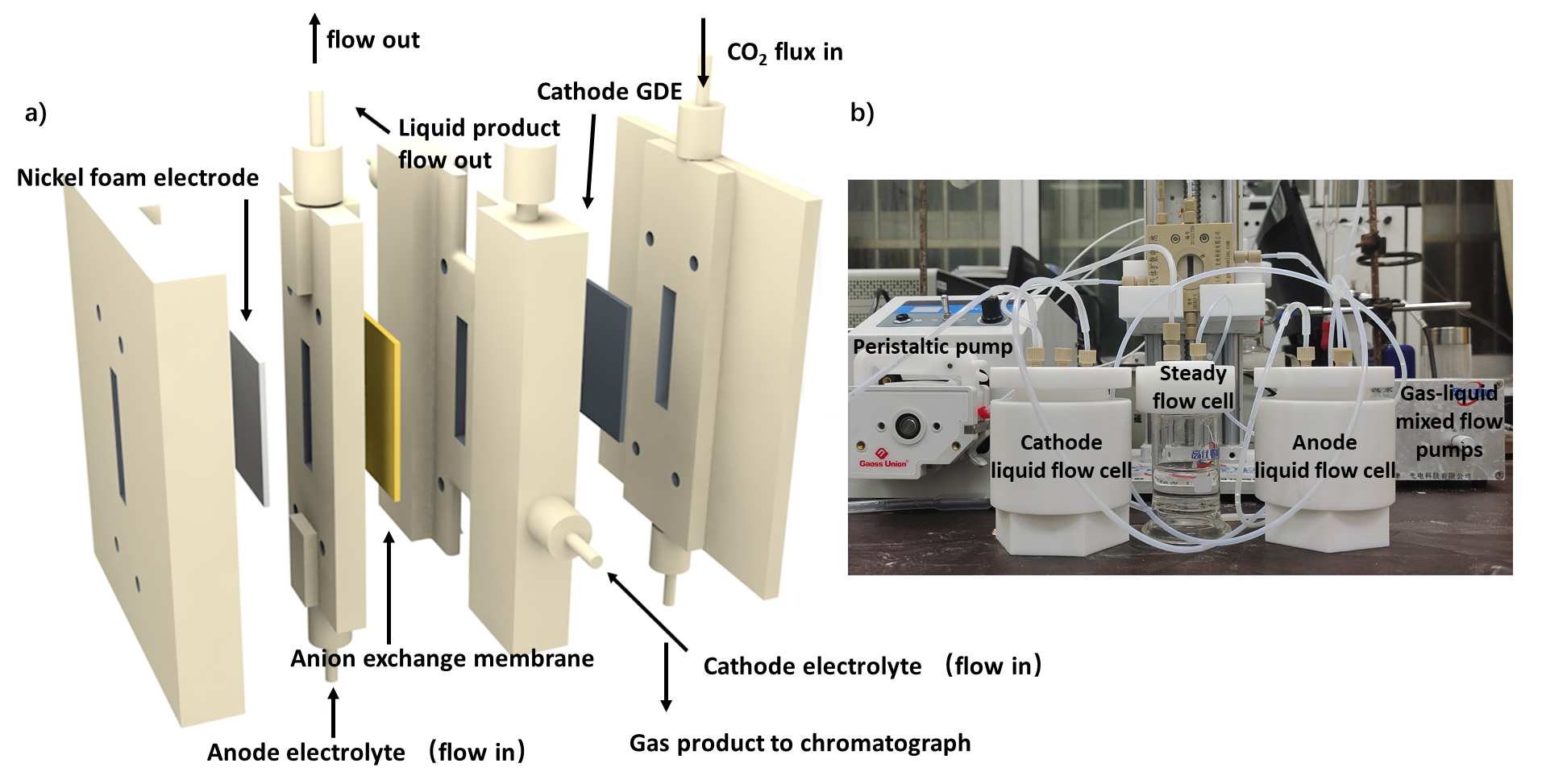


Figure S17. a) Schematic diagram depicting the structure and operation of the flow-cell, b) Photograph of the flow cell used for electrocatalytic CO_2_RR.


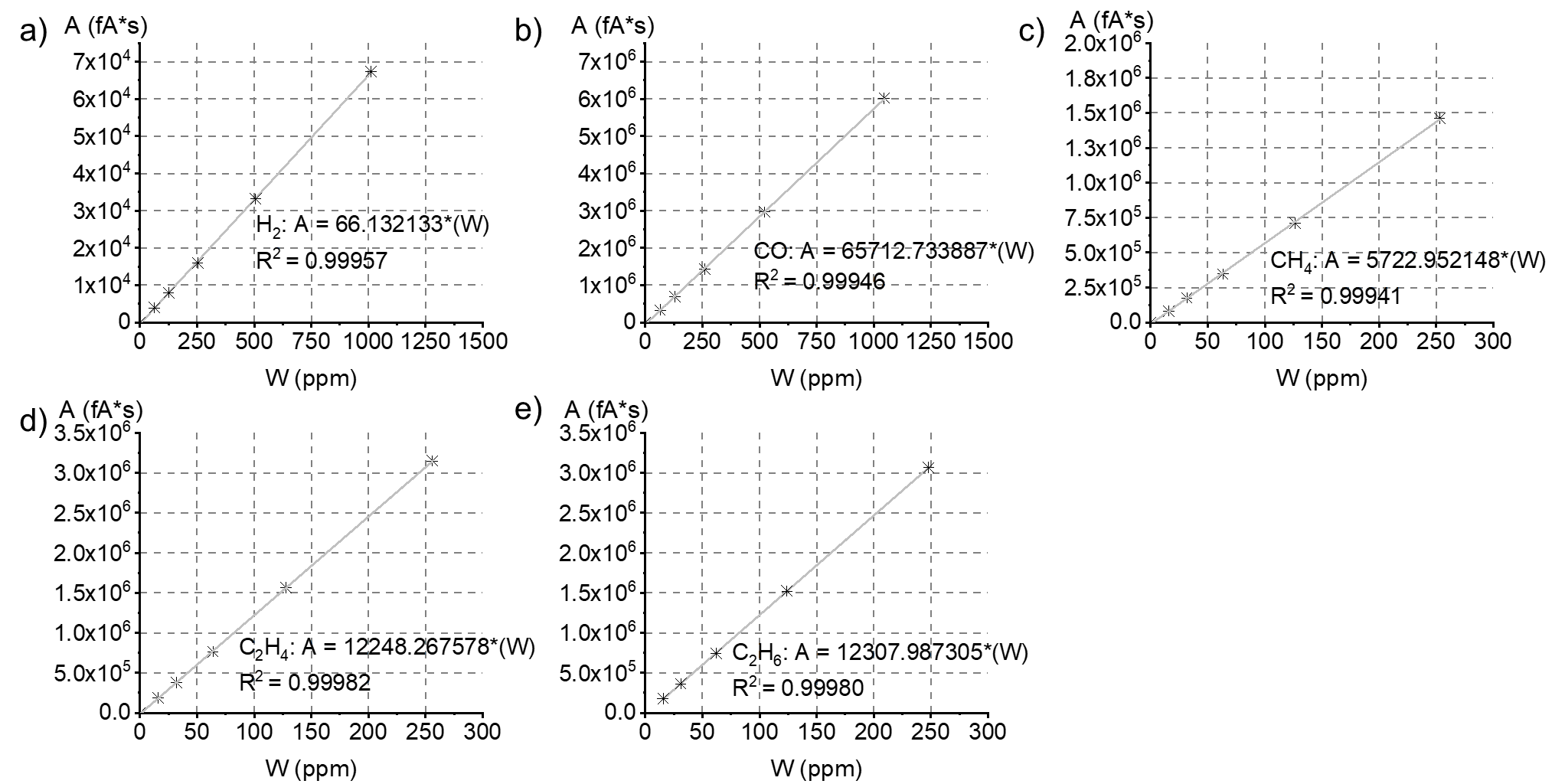


Figure S18. Standard curves for product detection by gas chromatography.


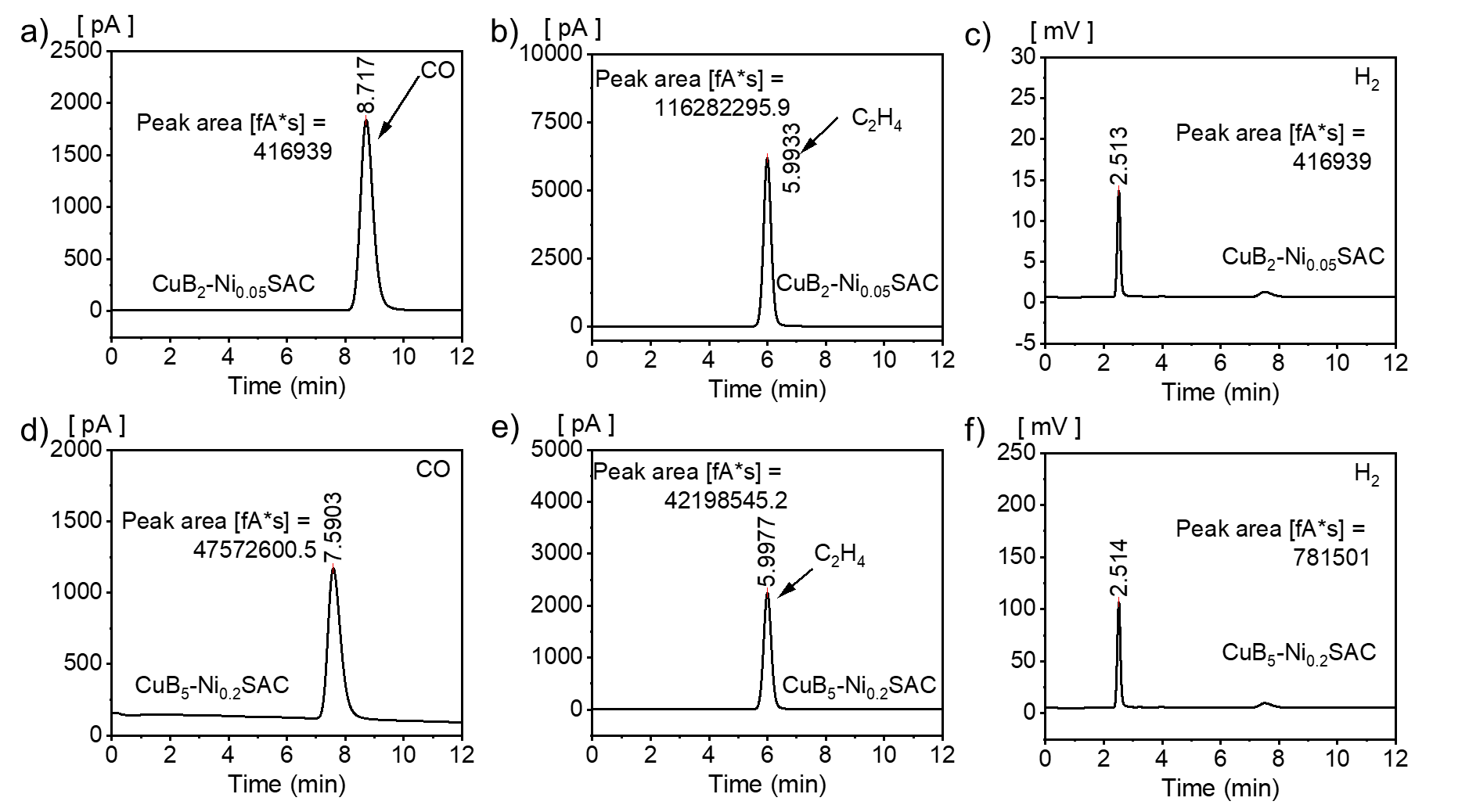


Figure S19. Gas chromatographic working curves of B-O-Cu2@Ni-SAC0.05 (a-c) and B-O-Cu2@Ni-SAC0.2 (d-f), respectively.


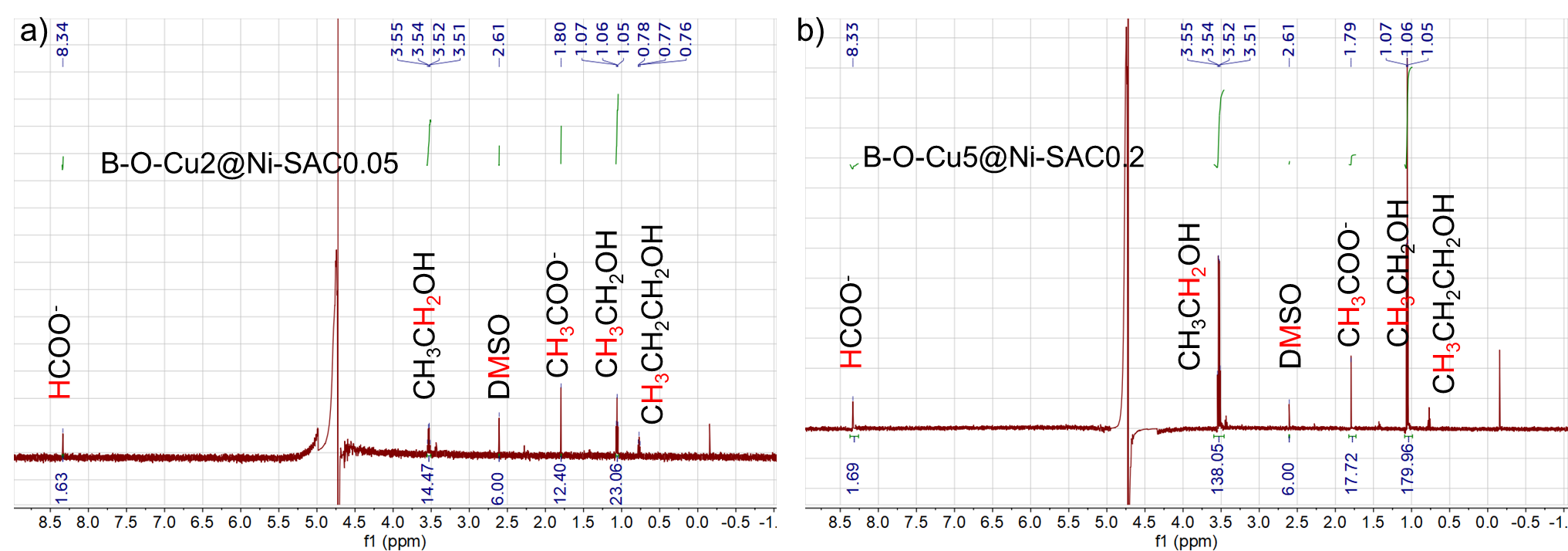


Figure S20. NMR spectra of liquid products. Representative ^1^H-NMR spectrum of catholyte after CO_2_RR on B-O-Cux*@Ni-SAC*y by applying -1.2 V vs. RHE in 0.5 M KHCO_3_. DMSO is used as an internal standard.


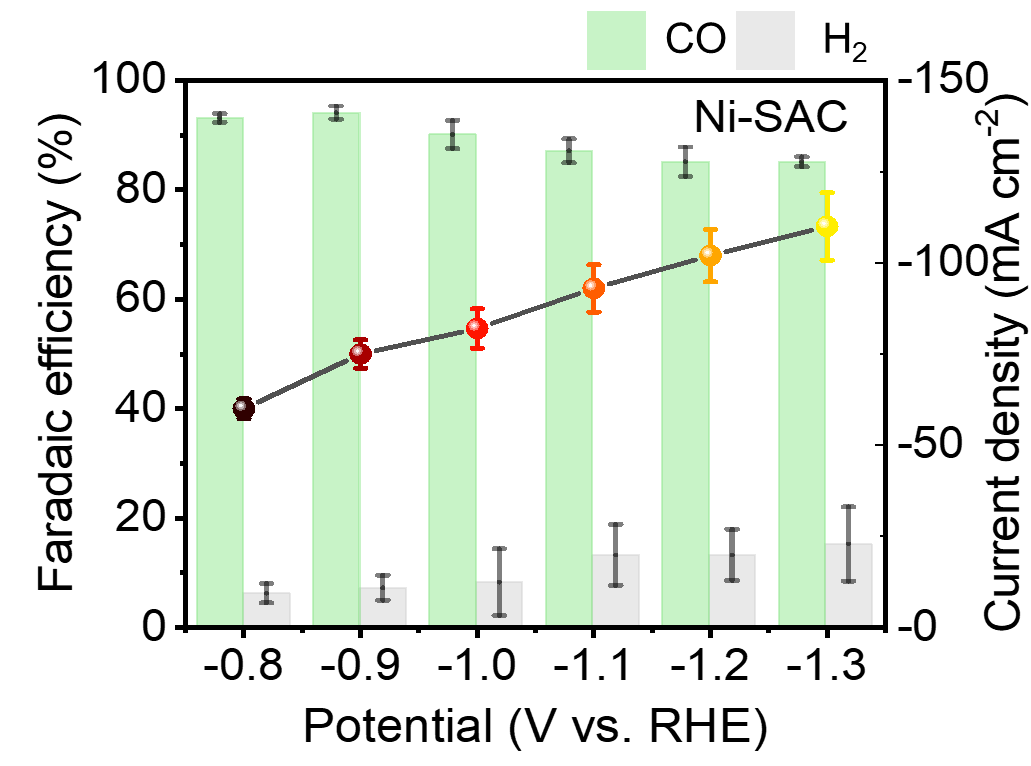


Figure S21. CO_2_RR performance of Ni-SAC in a flow cell with 0.5 M KHCO_3_ electrolyte.


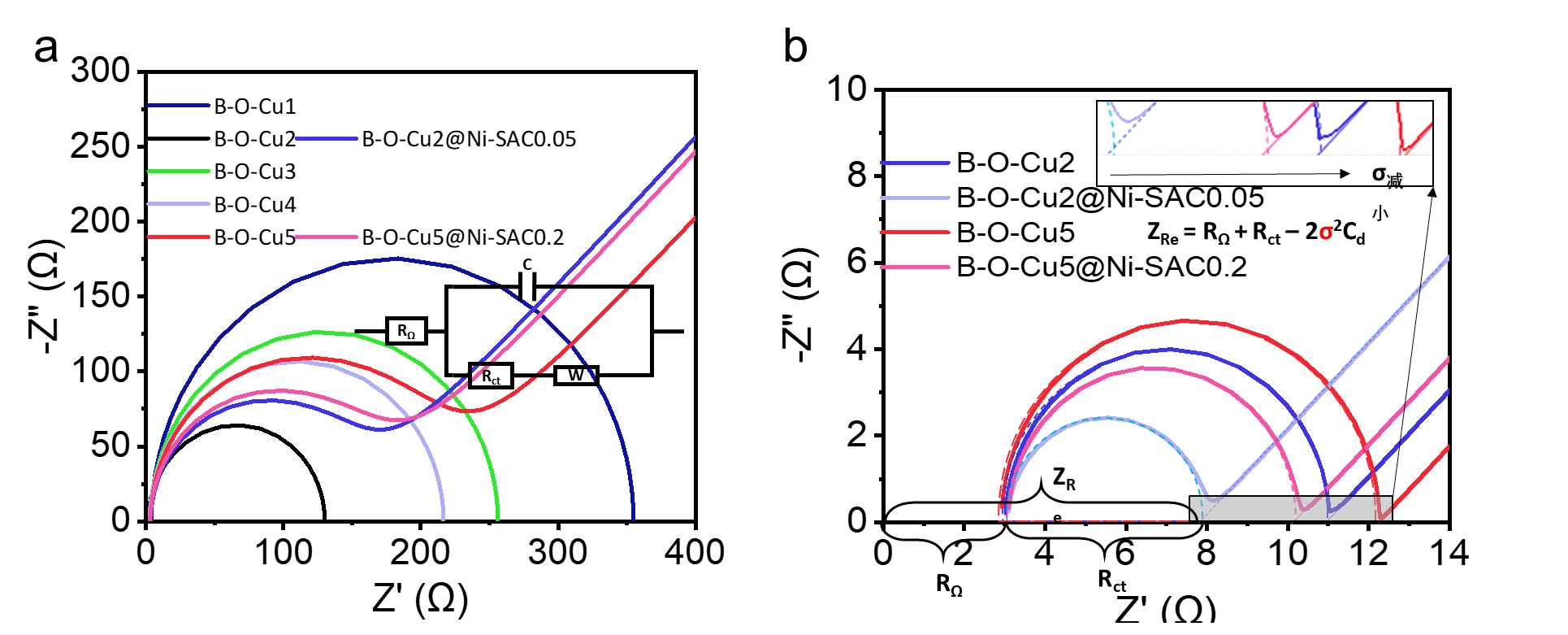


Figure S22. Electrochemical AC impedance Nyquist curves of hybrid catalysts with different B contents of B-O-Cux as well as partially doped Ni-SAC. Additionally, the EIS experiments for CO_2_RR were conducted at the open voltage (a) and -1.2 V vs. RHE (b). The and an amplitude of the sinusoidal voltage of 5 mV (in a frequency range of 1-10^6^ Hz)


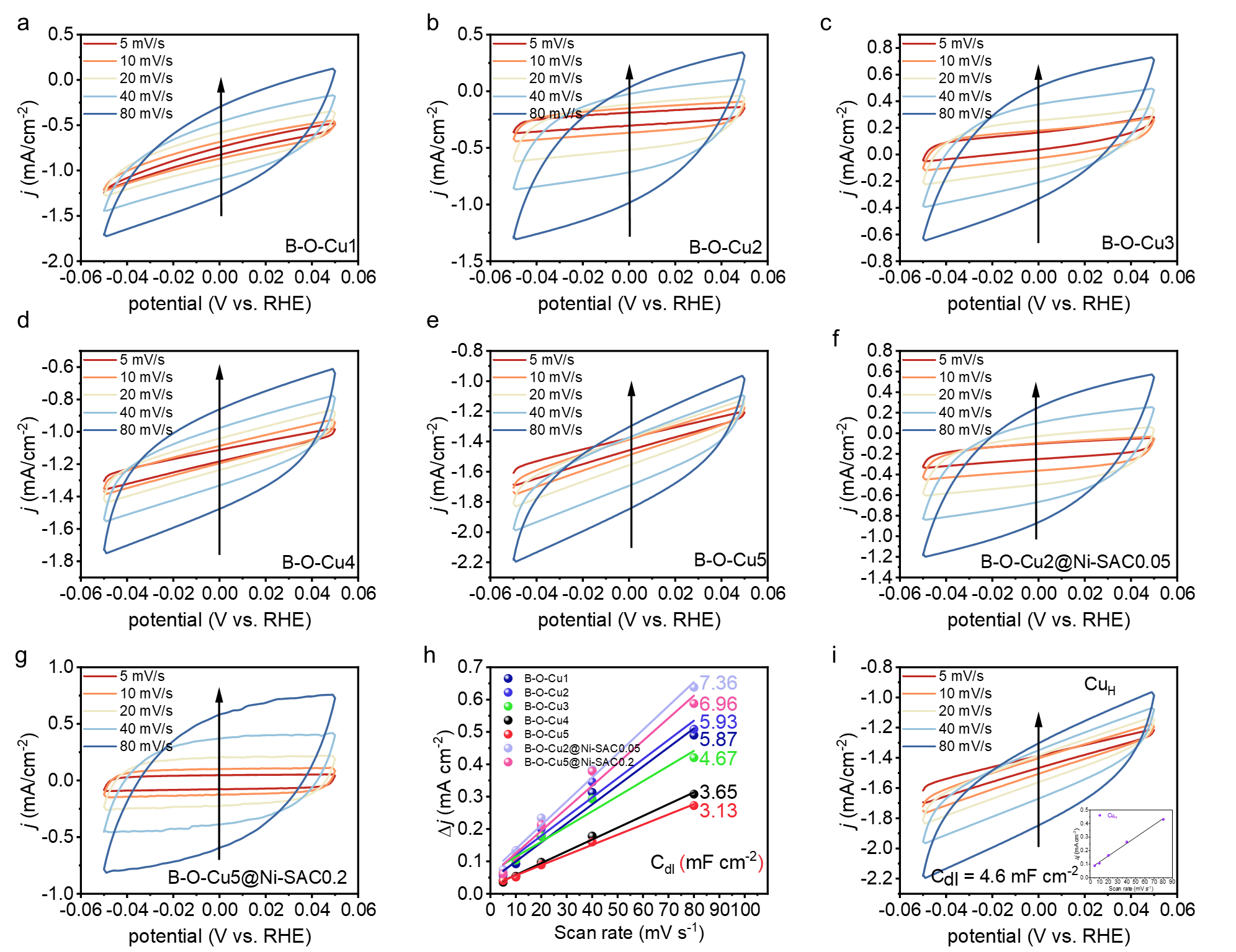


Figure S23. Electrochemically active surface area curves of hybrid catalysts with different B contents of B-O-Cux as well as partially doped Ni-SAC by cyclic voltammetry.


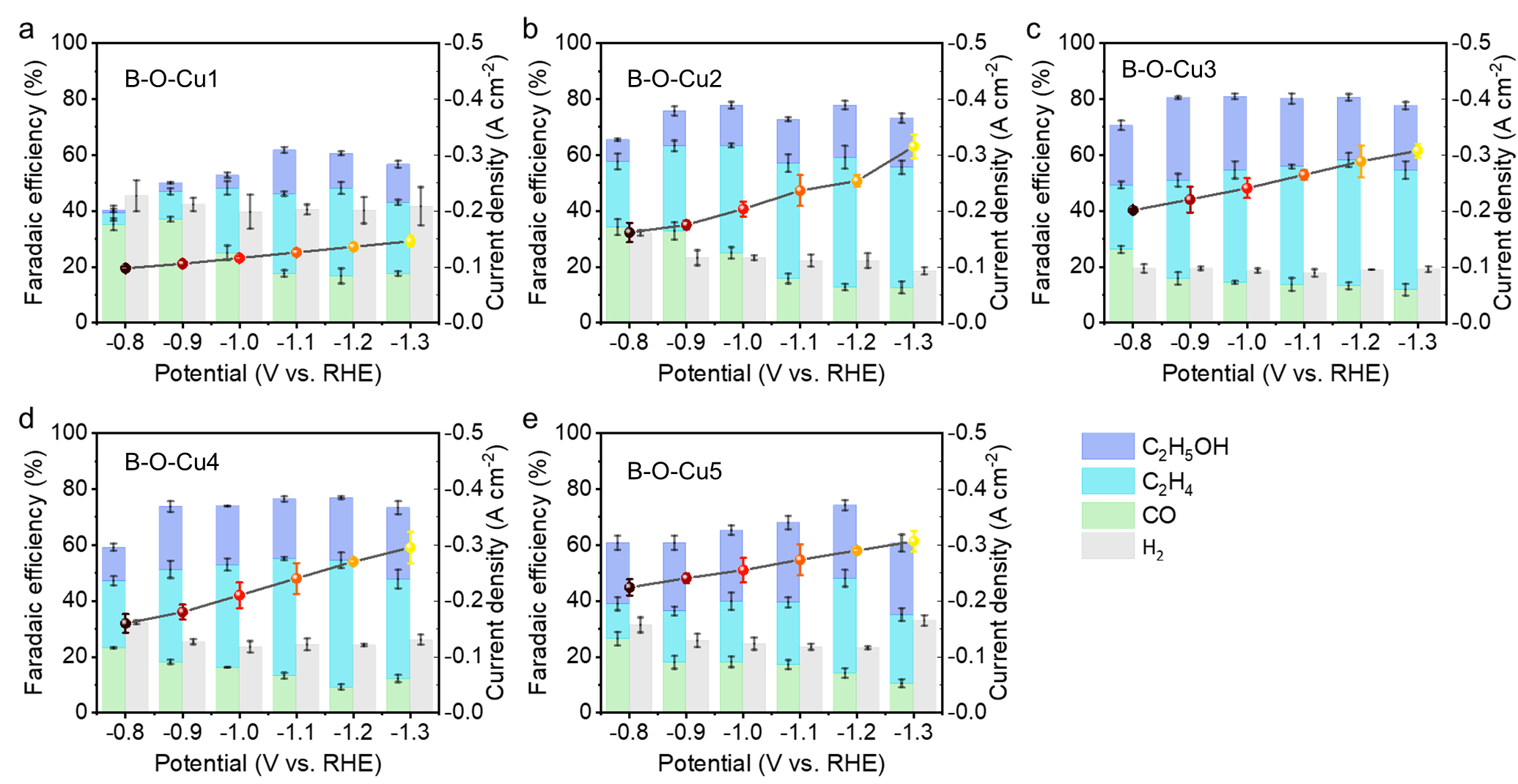


Figure S24. The CO_2_RR performance of unhybridized Ni-SAC with different contents of B-O-Cux at -0.8 to -1.3 V vs. RHE.


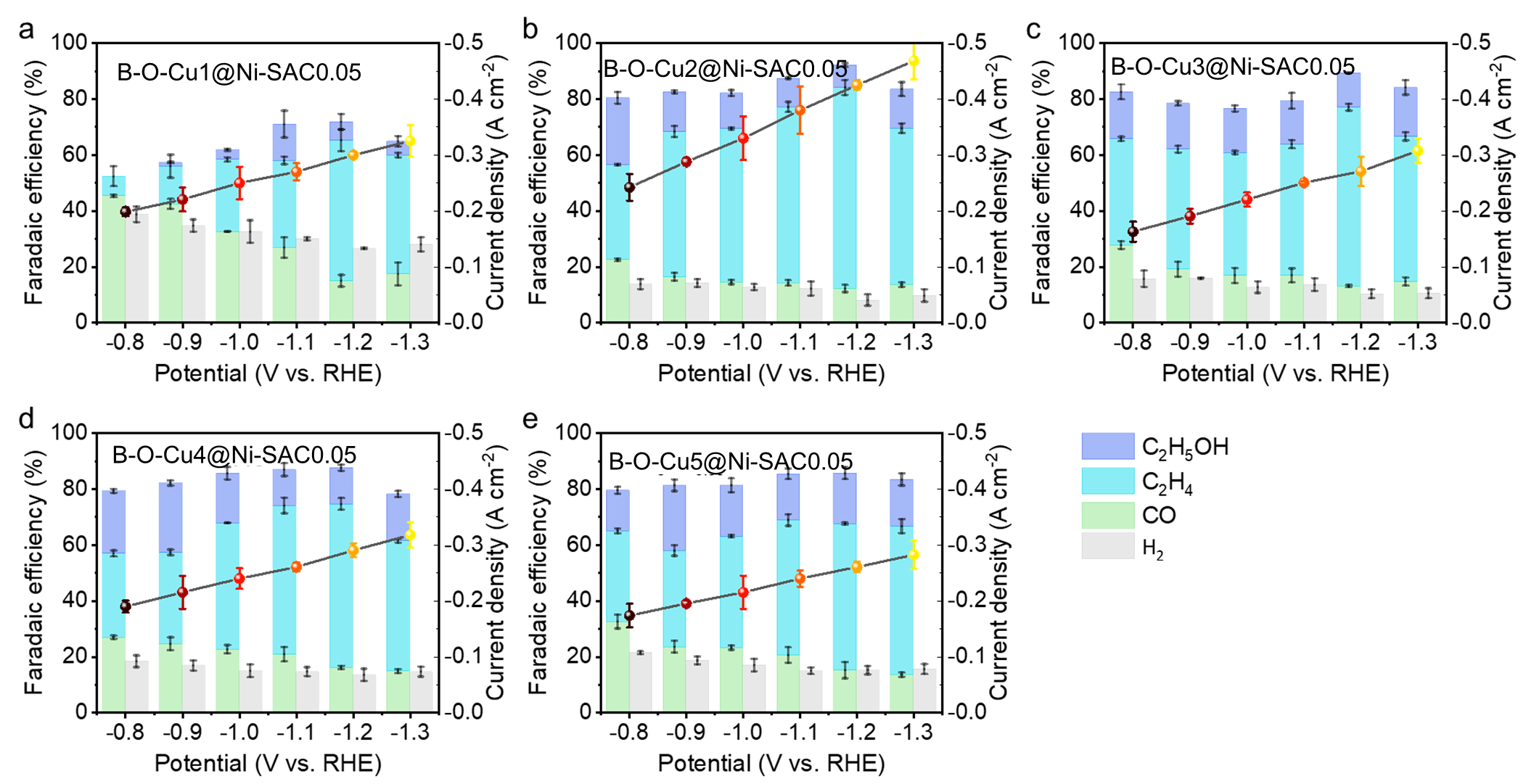


Figure S25.The mass ratio of Ni-SAC hybridized with different contents of B-O-Cux is 0.05, which corresponds to the CO_2_RR performance at -0.8 to -1.3 V vs. RHE.


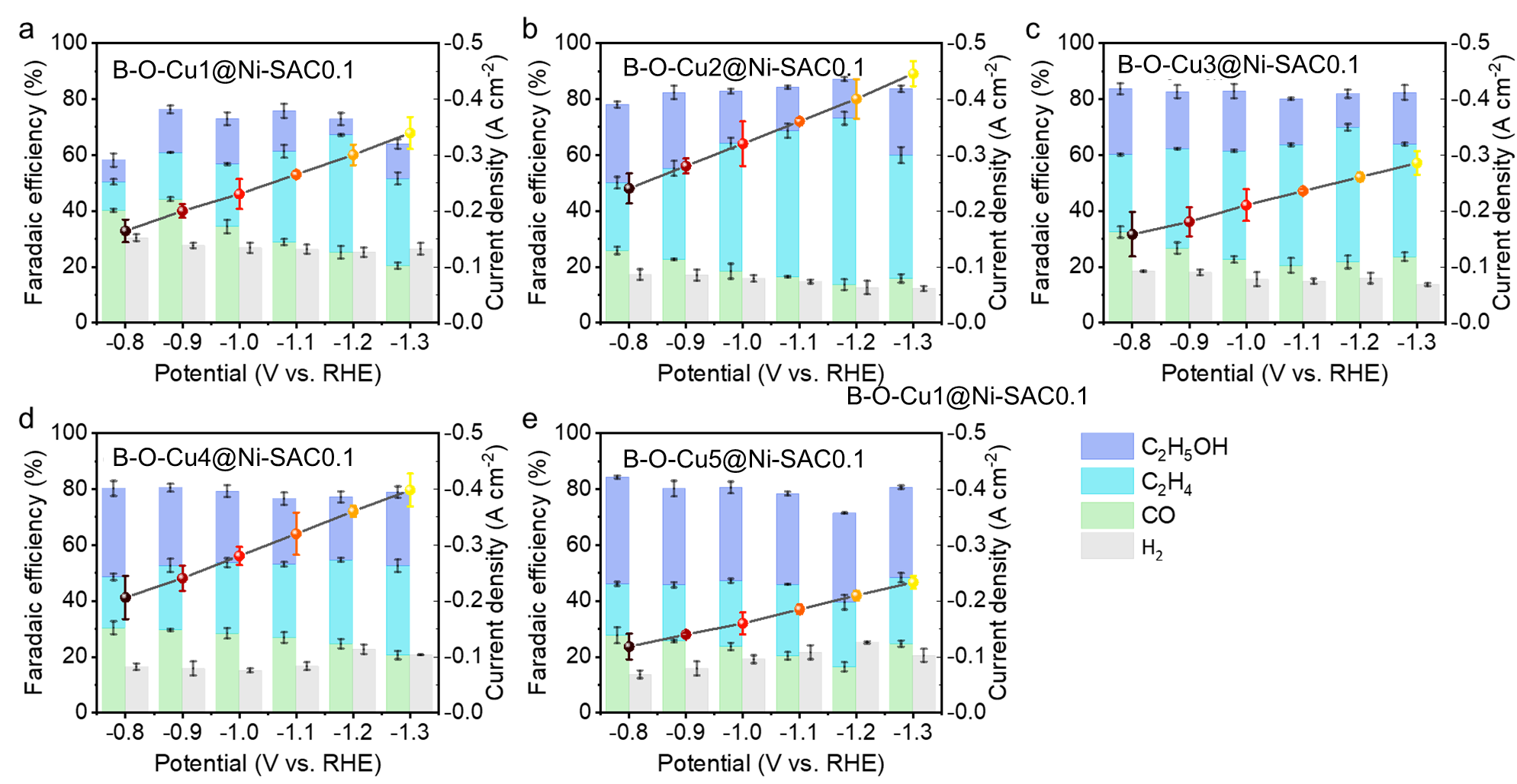


Figure S26.The mass ratio of Ni-SAC hybridized with different contents of B-O-Cux is 0.1, which corresponds to the CO_2_RR performance at -0.8 to -1.3 V vs. RHE.


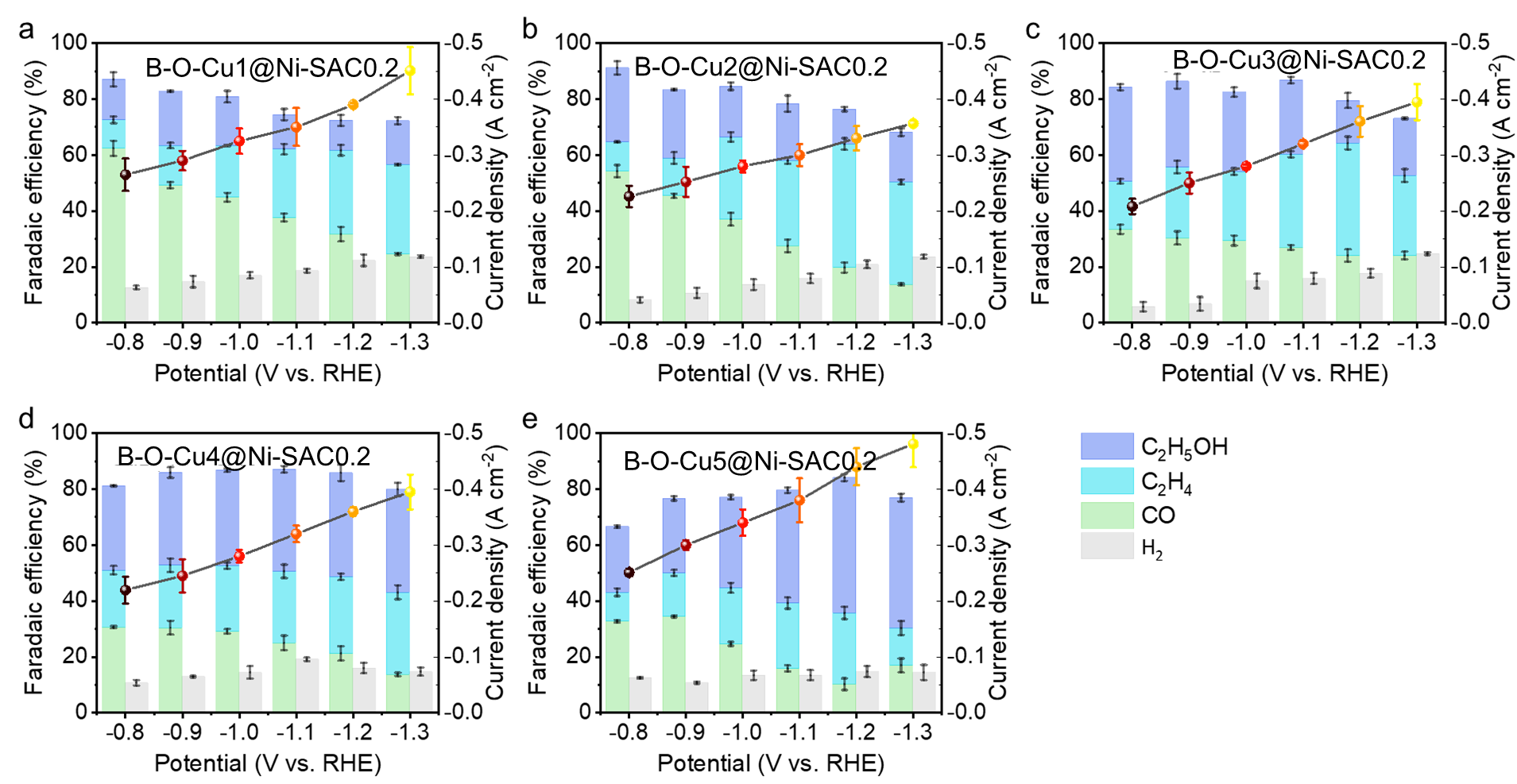


Figure S27.The mass ratio of Ni-SAC hybridized with different contents of B-O-Cux is 0.2, which corresponds to the CO_2_RR performance at -0.8 to -1.3 V vs. RHE.


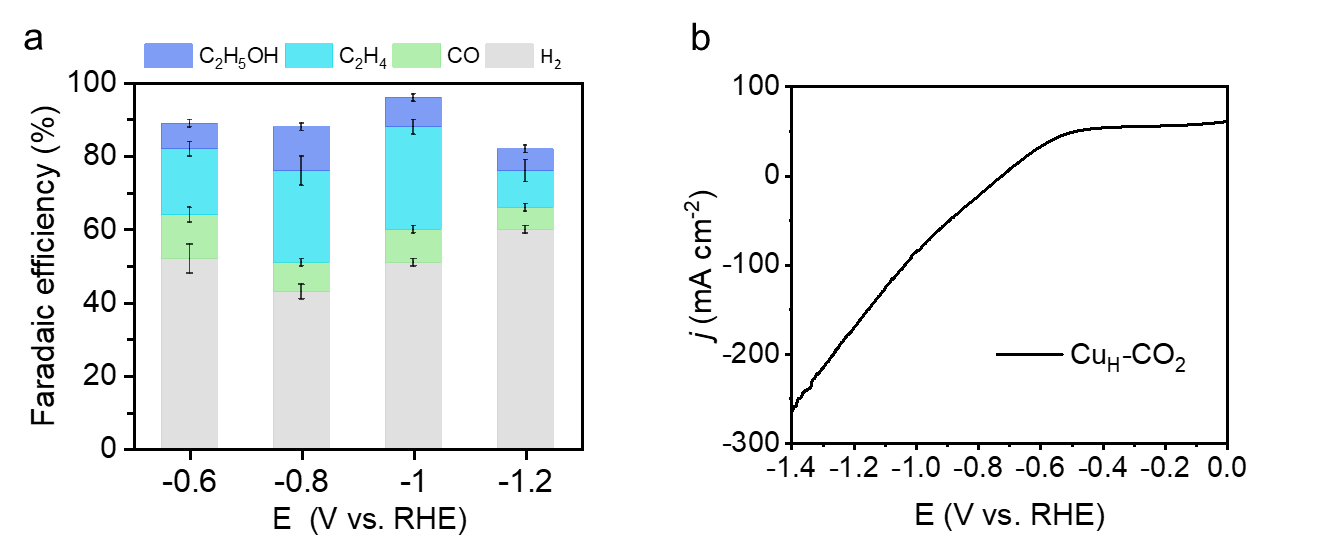


Figure S28. a) the FE performance at -0.6 to -1.2 V vs. RHE of Cu_H_ in 0.5 M CO_2_- saturated KHCO_3,_ and corresponding b) Linear voltametric curves.


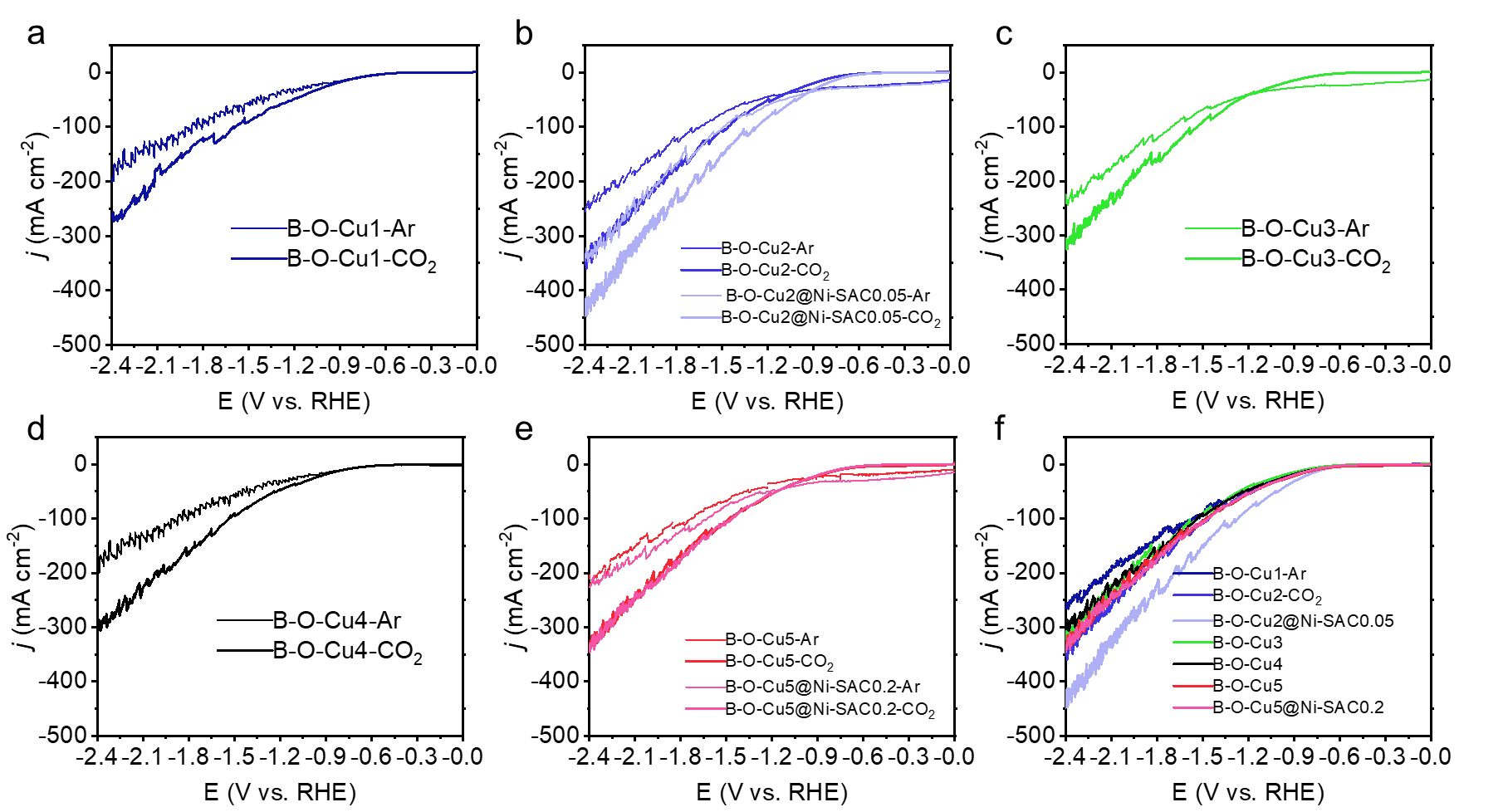


Figure S29. Linear voltammetric curves of hybrid catalysts with different B contents of B-O-Cux as well as partially doped Ni-SAC under different atmospheres. The CO_2_RR performance of the synthesized catalysts was tested in a flow cell in 0.5 M KHCO_3_ electrolyte.


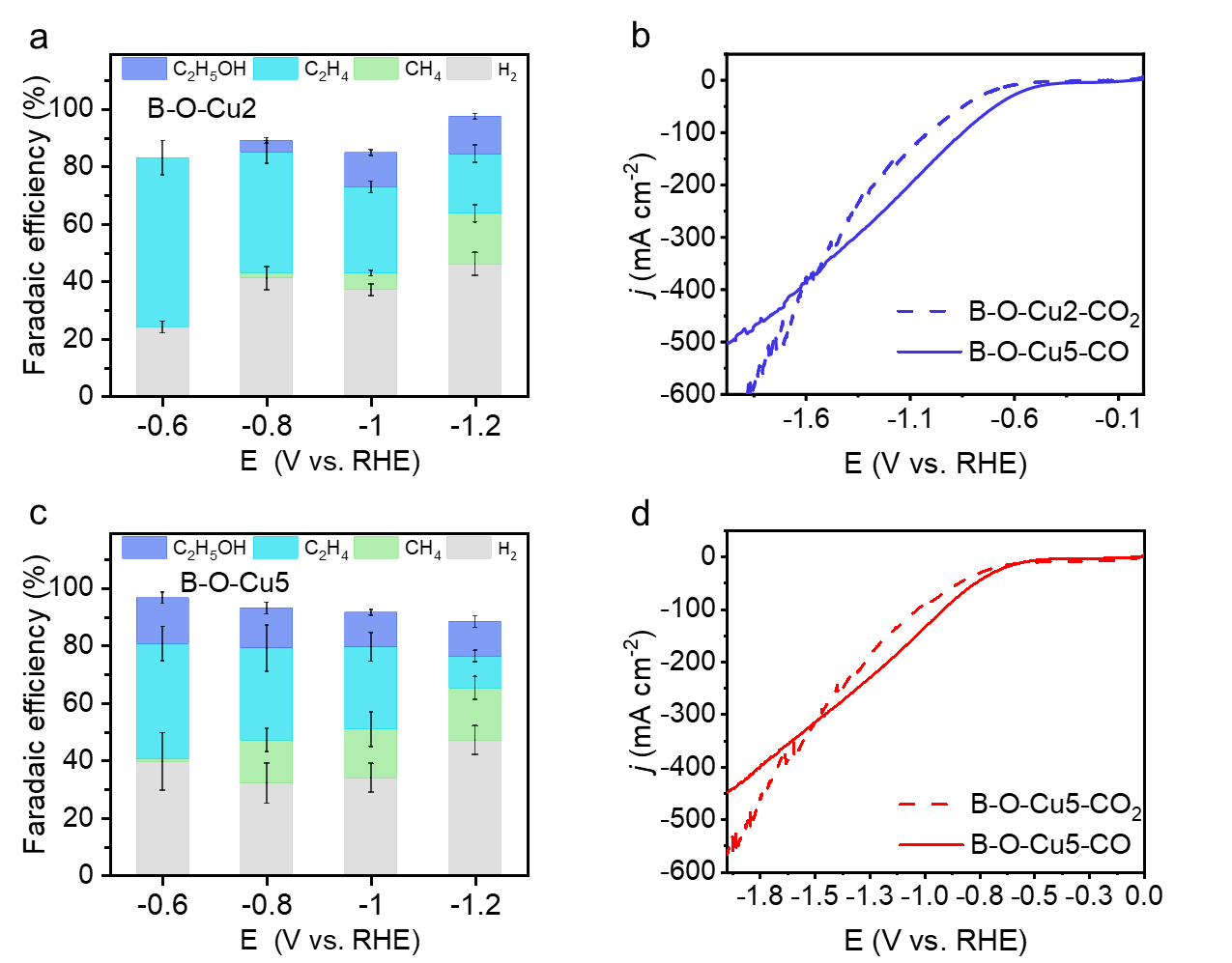


Figure S30. a, c)the FE performance at -0.6 to -1.2 V vs. RHE of B-O-Cu2 in 0.5 M CO- saturated KHCO_3,_ and corresponding b, d) Linear voltametric curves.


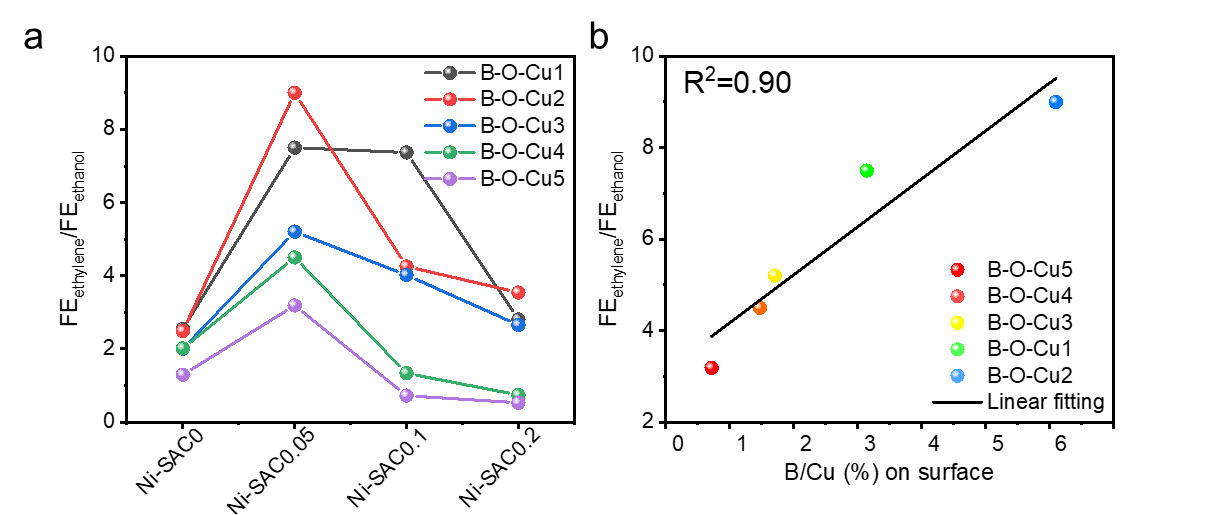


Figure S31. a) CO_2_RR Performance with Varying Ni-SAC Assembly Ratios in Different B-Doped Samples. b) The fitted curve of the ethylene-to-ethanol conversion ratio with the increasing surface B/Cu atomic ratio

Figure S32. Stability of B-O-Cu2 tested by pulse voltage method at -1.2 V vs. RHE.


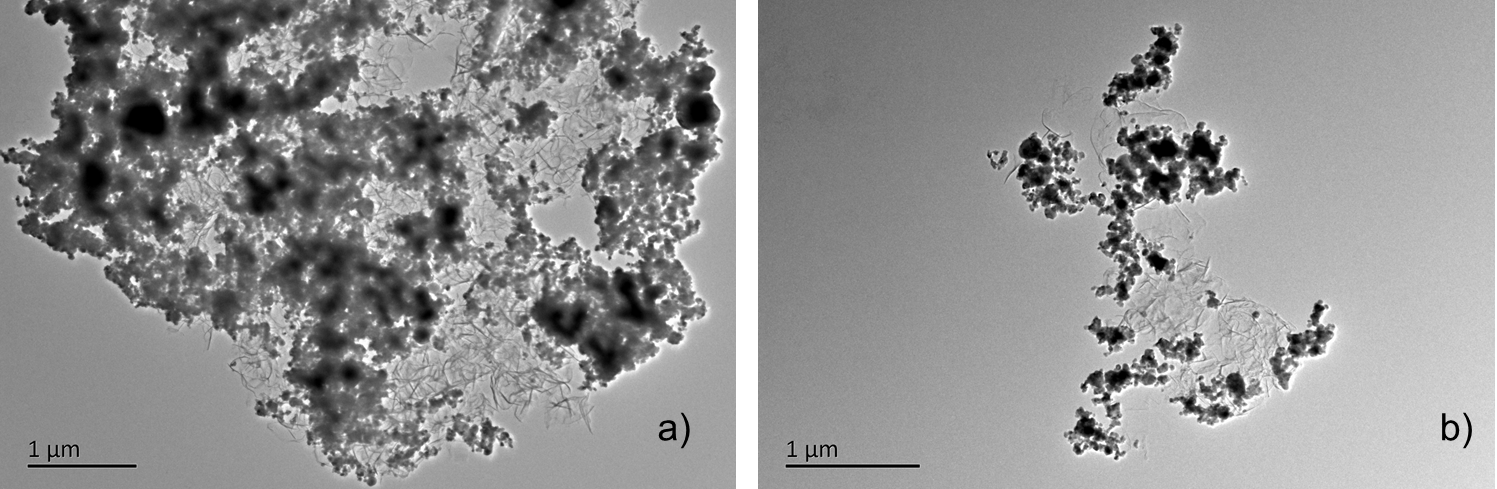
Figure S33. TEM images of B-O-Cu2@Ni-SAC0.05 (a) and B-O-Cu5@Ni-SAC0.2 (b) after 1 hour of electrolysis.

Figure S34.The near-edge first-order derivative diagram of Cu K-edge Survey of B-O-Cu2.


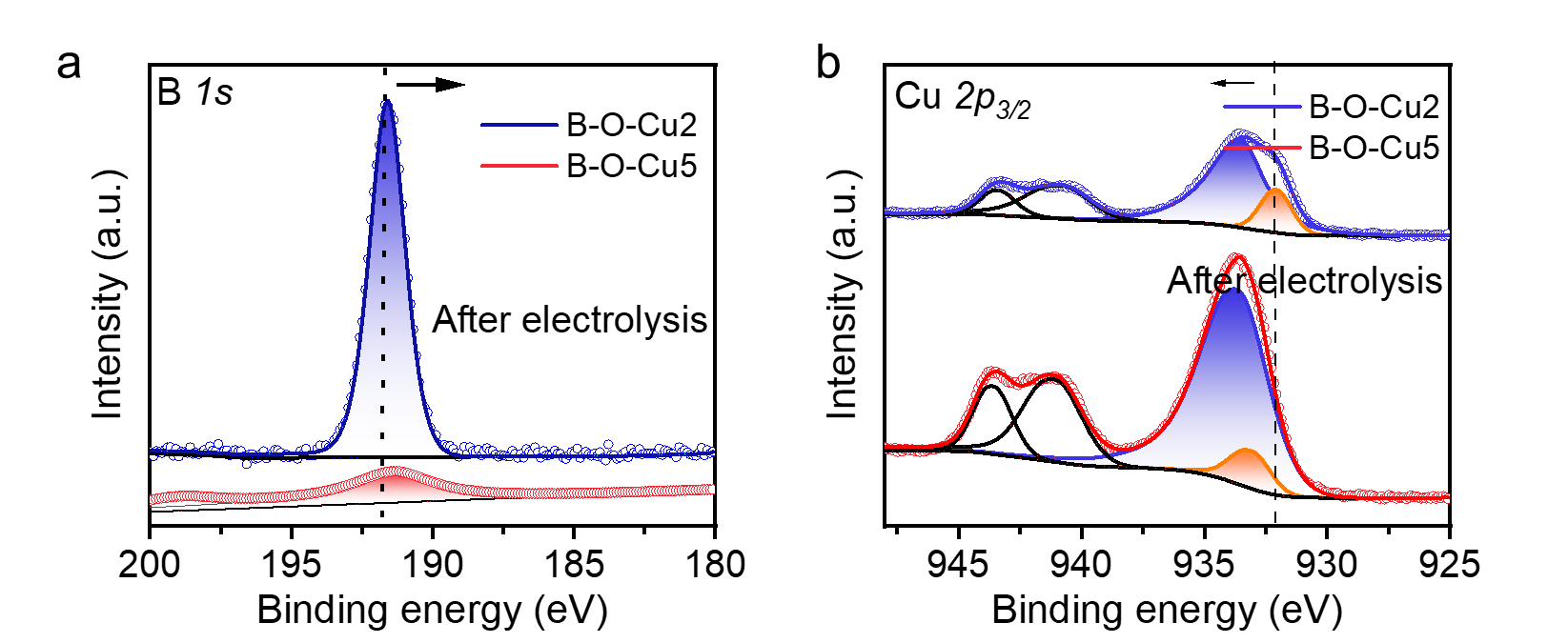


Figure S35. XPS spectra of B-O-Cu2 (a) and B-O-Cu5 (b) after 1 hour of electrolysis.


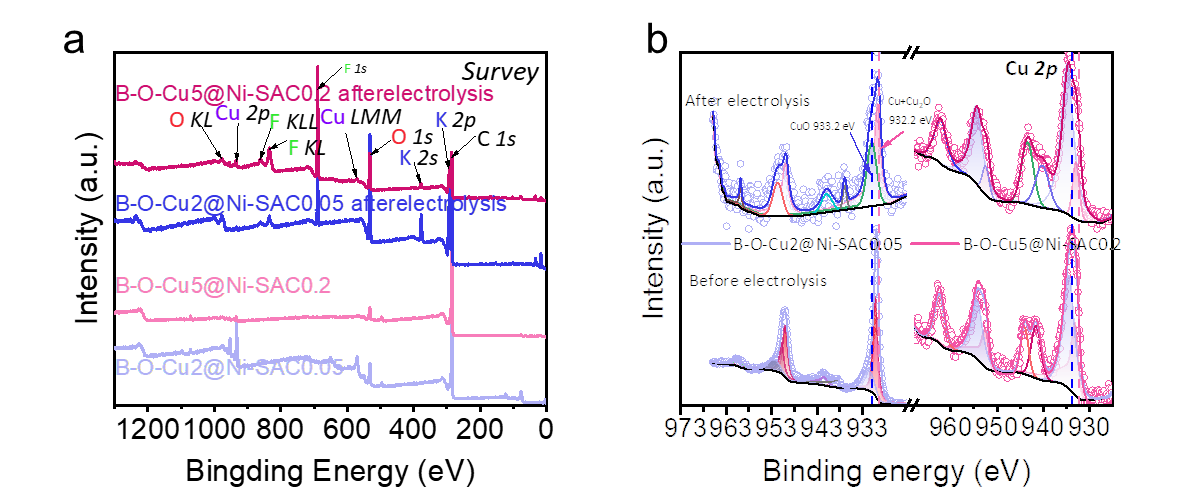


Figure S36. XPS spectra of B-O-Cu2@Ni-SAC0.05(a) and B-O-Cu5@Ni-SAC0.2 (b) after 1 hour of electrolysis.

Figure S37. Solid-state Raman profiles of Cu_2_O and B-O-Cu2 samples.


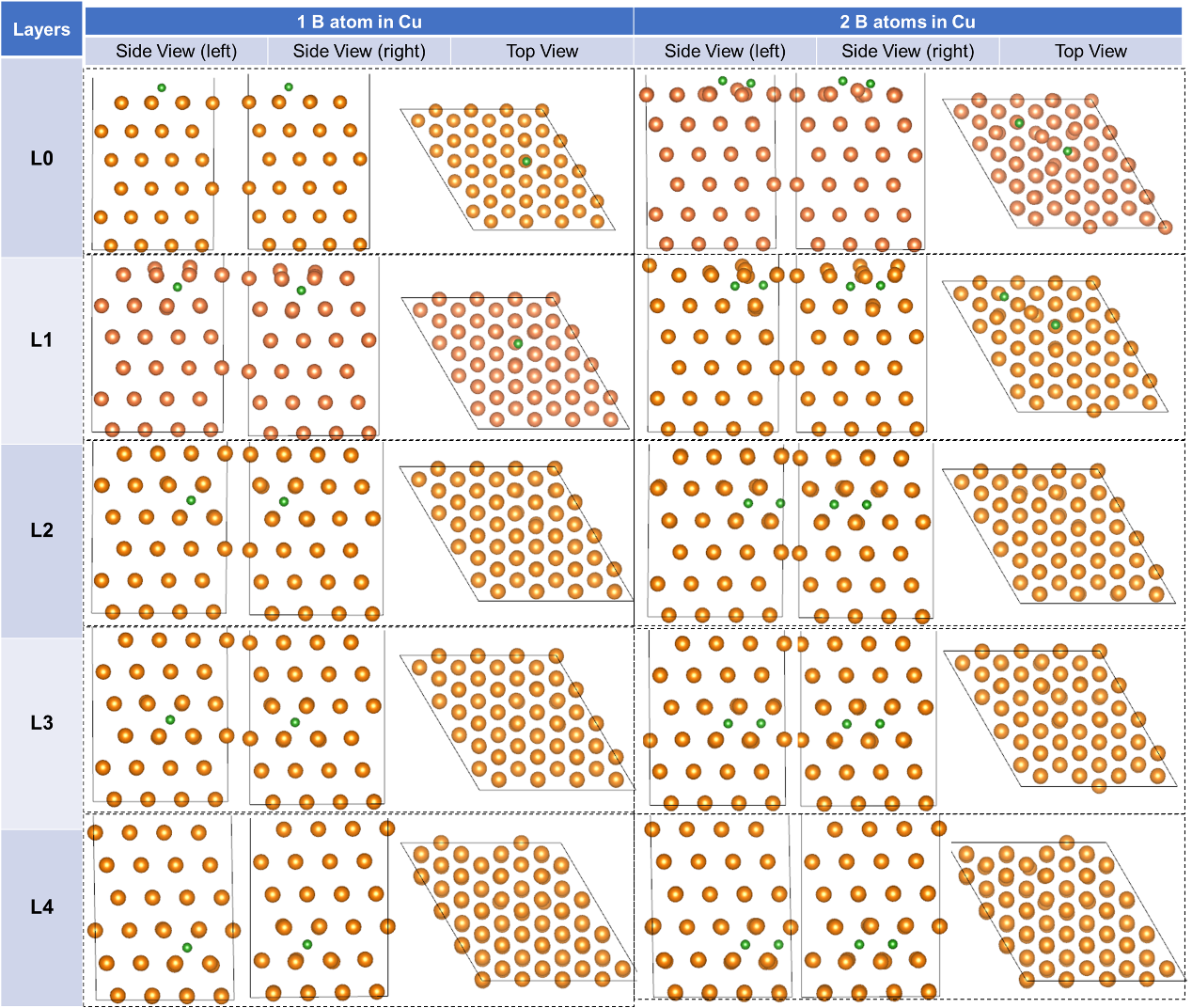
 Figure S38. Conformations of 1 B atom and 2 B atoms in different layers of the Cu crystalline phase.


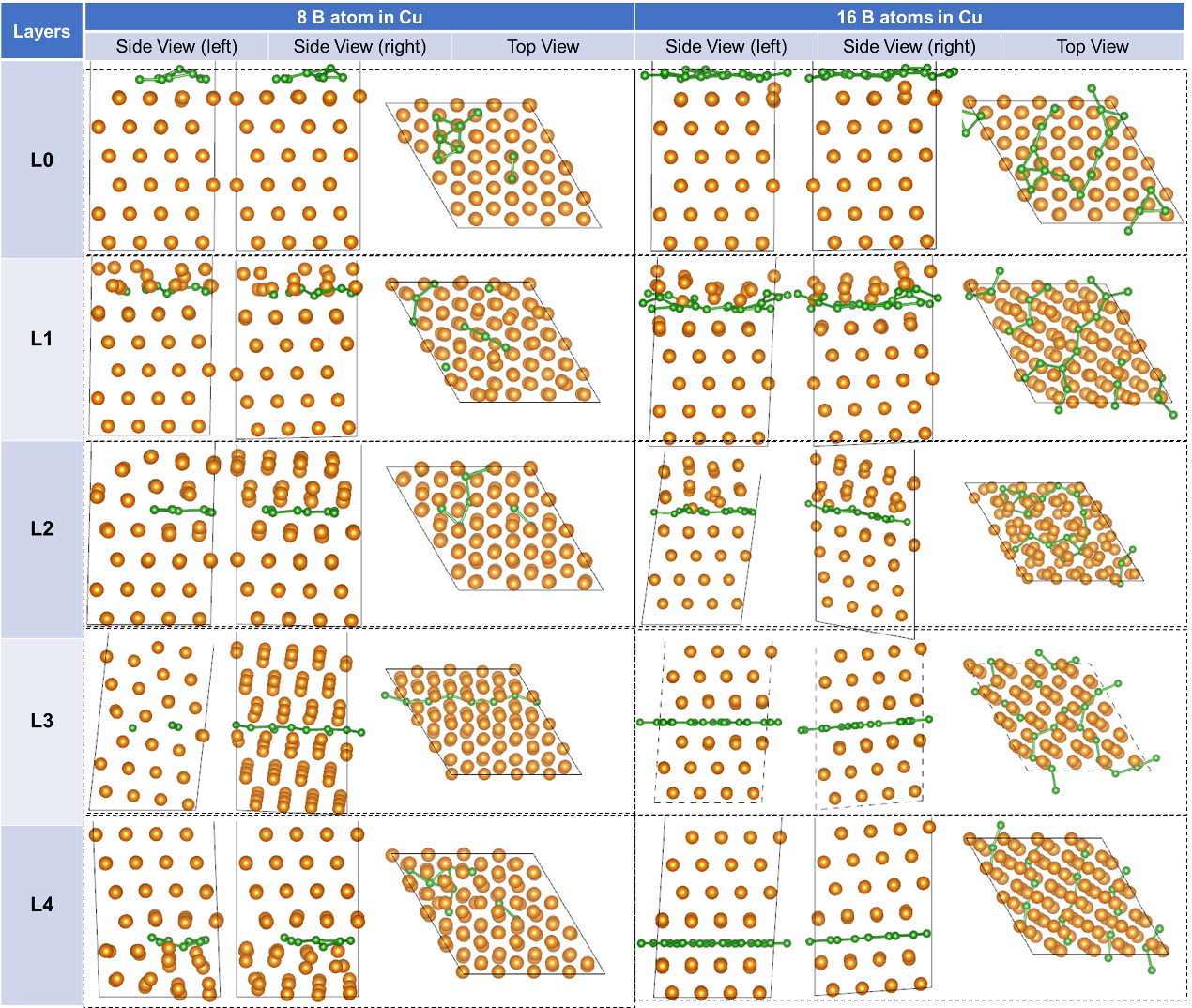
Figure S39. Configurations of 8 B atom and 16 B atoms in different layers of the Cu crystalline phase.


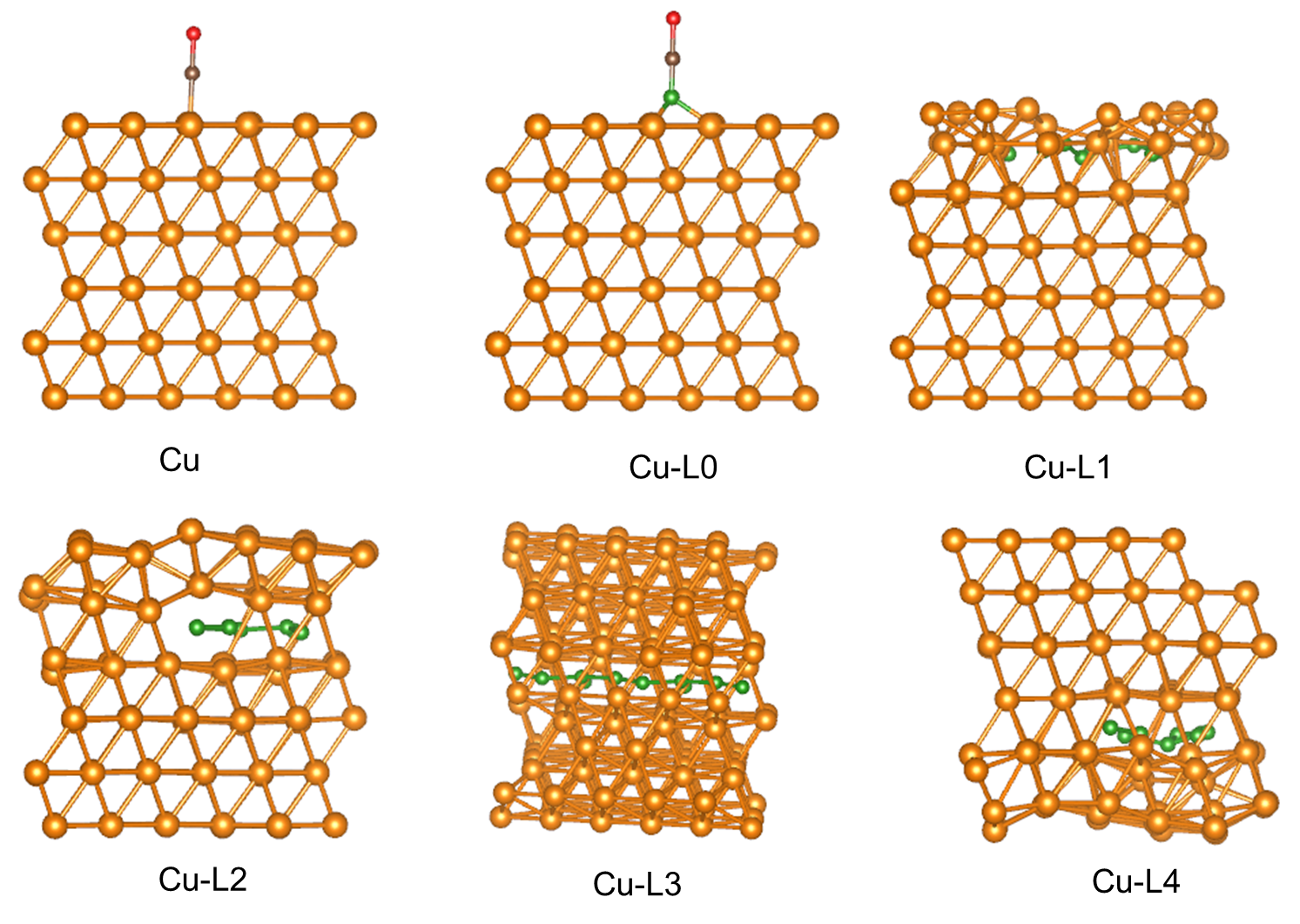


Figure S40. Catalytic configuration for calculating the adsorption energy of *CO.


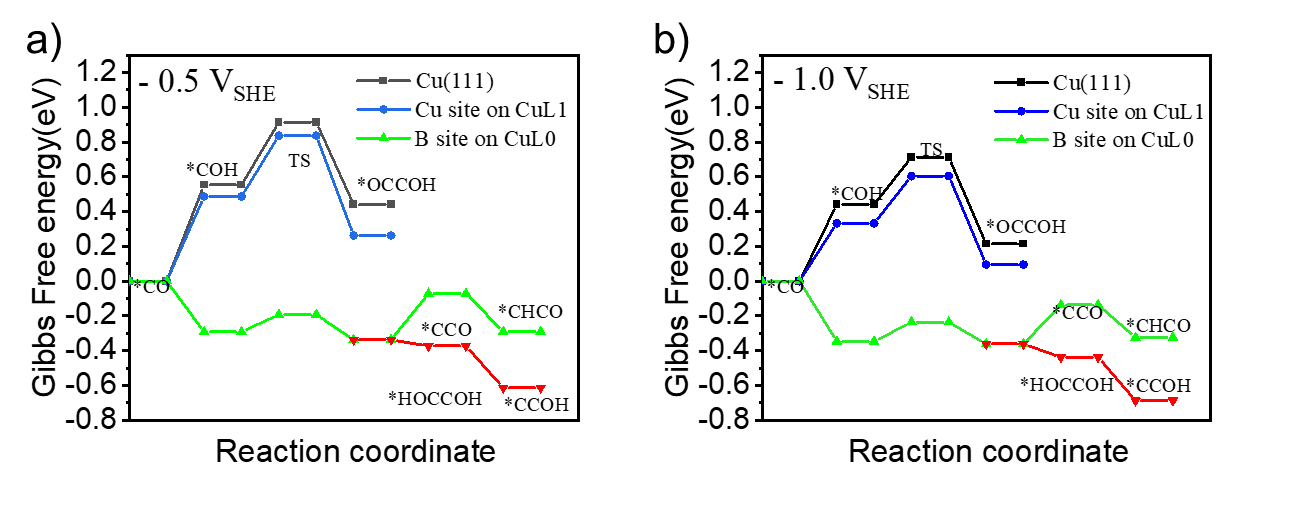


Figure S41. Gibbs Free energy diagrams for C-C coupling over different catalysts at U=-0.5（A） and -1.0 V（B） vs the SHE calculated using the CEP model. Reaction free energies are in eV.

Table S1 B contents in samples synthesized using different amount of CuCl_2_.

| Samples | No. | | B(mg/L) | Cu(mg/L) | B/Cu(atomic ratio in ICP) | B/Cu(atomic ratio in XPS) |
| --- | --- | --- | --- | --- | --- | --- |
| B-O-Cu1 | | #1 | 0.14987 | 82.92678 | 0.0107 | 2.13 |
|  |  | #2 | 0.16014 | 85.72207 | 0.0110 | 3.57 |
|  |  | #3 | 0.17076 | 88.51735 | 0.0114 | 3.73 |
| B-O-Cu2 | | #1 | 0.16620 | 82.00043 | 0.012 | 5.51 |
|  |  | #2 | 0.17759 | 84.76449 | 0.0124 | 6.23 |
|  |  | #3 | 0.18937 | 87.52855 | 0.0128 | 6.56 |
| B-O-Cu3 | | #1 | 0.25867 | 78.87073 | 0.0194 | 0.47 |
|  |  | #2 | 0.27954 | 81.52929 | 0.0203 | 1.95 |
|  |  | #3 | 0.29807 | 84.18786 | 0.0209 | 2.69 |
| B-O-Cu4 | | #1 | 0.28234 | 76.71522 | 0.0217 | 0.36 |
|  |  | #2 | 0.30158 | 79.30113 | 0.0225 | 1.73 |
|  |  | #3 | 0.31811 | 81.88703 | 0.0230 | 2.32 |
| B-O-Cu5 | | #1 | 0.35004 | 70.90109 | 0.0292 | 0.3 |
|  |  | #2 | 0.37403 | 73.29101 | 0.0302 | 0.41 |
|  |  | #3 | 0.39882 | 75.68094 | 0.0312 | 1.44 |

Table S2. Time-dependent B/Cu-ICP testing in B-O-Cu2 and B-O-Cu5 sample.

| Samples | | Dissolution time | B(mg/L) | Cu(mg/L) | B/Cu(atomic ratio%) |
| --- | --- | --- | --- | --- | --- |
| B-O-Cu2 | 10s | | 0.04358 | 6.617 | 3.9 |
|  | 120s | | 0.05032 | 9.672556 | 3.08 |
|  | 300s | | 0.05523 | 12.92589 | 2.53 |
|  | 600s | | 0.05043 | 18.89885 | 1.58 |
|  | 1800s | | 0.06546 | 19.38 | 2 |
| B-O-Cu5 | 10s | | 0.01442 | 5.617 | 1.52 |
|  | 120s | | 0.03585 | 11.473 | 1.85 |
|  | 300s | | 0.06220 | 13.152 | 2.8 |
|  | 600s | | 0.07980 | 14.765 | 3.2 |
|  | 1800s | | 0.09163 | 16.44 | 3.3 |

Table S3 B contents in samples after 1 hour of electrolysis.

| Samples after eletrolysis | B-O-Cu2 | B-O-Cu5 |
| --- | --- | --- |
| B/Cu (atomic ratio %) in ICP | 1.5 | 3.3 |
| B/Cu (%) in XPS | 8.56 | 0.38 |

Table S4 Curvefit Parametes^a^ for Cu K-edge EXAFS for B-O-Cu2 sample.

| Path | *d*^b^/Å | *N(±*ΔN*)*^c^ | *R* (ΔR) / Å | σ^2^/ Å^2^ |
| --- | --- | --- | --- | --- |
| [Cu]Cu-Cu | 2.556 | 2.33(±1) | 2.58(0.05) | 0.0055 |
| [Cu_2_O]Cu-O1 | 1.841 | 1.29(±0.6) | 1.90(0.02) | 0.0051 |

^a^ S_0_^2^ was fixed as 0.92 (From the same experimental group of Cu standard sample). Δ*E*_0_ was refined as a segregate fit parameter, returning a value of 9.328 eV. Data ranges: 3 ≤ k ≤ 10.5 Å^-1^, 1.0 < R < 3.0 Å. The number of variable parameters is 7, out of a total of 8.75 independent data points. R factor for this fit is 1.4%. ^b^ The distances for Cu-O and Cu-Cu are from the crystal structure of Cu, Cu_2_O.

Table S5. Curvefit Parameters^a^ for Cu K-edge EXAFS for B-O-Cu5 sample

| Path | *d*/Å | *N(±*ΔN*)*^c^ | *R* (ΔR) / Å | σ^2^/ Å^2^ |
| --- | --- | --- | --- | --- |
| [Cu]Cu-Cu | 2.556 | 1.92(±0.8) | 2.58(0.06) | 0.0037 |
| [Cu_2_O]Cu-O | 1.841 | 1.44(±0.37) | 1.88(0.13) | 0.0036 |

^a^ S_0_^2^ was fixed as 0.92 (From the same experimental group of Cu standard sample). Δ*E*_0_ was refined as a segregate fit parameter, returning a value of (5 ± 2) eV. Data ranges: 3 ≤ k ≤ 10.5 Å^-1^, 1.0 < R < 3.0 Å. The number of variable parameters is 7, out of a total of 9.31 independent data points. R factor for this fit is 2.4%.
